# Validation of DE50-MD dogs as a model for the brain phenotype of Duchenne muscular dystrophy

**DOI:** 10.1242/dmm.049291

**Published:** 2022-03-02

**Authors:** Abbe H. Crawford, John C. W. Hildyard, Sophie A. M. Rushing, Dominic J. Wells, Maria Diez-Leon, Richard J. Piercy

**Affiliations:** 1 Comparative Neuromuscular Diseases Laboratory, Department of Clinical Science and Services, Royal Veterinary College, London NW1 0TU, UK; 2 Department of Comparative Biomedical Sciences, Royal Veterinary College, London NW1 0TU, UK; 3 Pathobiology and Population Sciences, Royal Veterinary College, London AL9 7TA, UK

**Keywords:** Duchenne muscular dystrophy, Brain, Cognitive, Dog, Animal model

## Abstract

Duchenne muscular dystrophy (DMD), a fatal musculoskeletal disease, is associated with neurodevelopmental disorders and cognitive impairment caused by brain dystrophin deficiency. Dog models of DMD represent key translational tools to study dystrophin biology and to develop novel therapeutics. However, characterisation of dystrophin expression and function in the canine brain is lacking. We studied the DE50-MD canine model of DMD that has a missense mutation in the donor splice site of exon 50. Using a battery of cognitive tests, we detected a neurocognitive phenotype in DE50-MD dogs, including reduced attention, problem solving and exploration of novel objects. Through a combination of capillary immunoelectrophoresis, immunolabelling, quantitative PCR and RNAScope *in situ* hybridisation, we show that regional dystrophin expression in the adult canine brain reflects that of humans, and that the DE50-MD dog lacks full-length dystrophin (Dp427) protein expression but retains expression of the two shorter brain-expressed isoforms, Dp140 and Dp71. Thus, the DE50-MD dog is a translationally relevant pre-clinical model to study the consequences of Dp427 deficiency in the brain and to develop therapeutic strategies for the neurological sequelae of DMD.

## Abstract (precis)

**Summary:** The DE50-MD canine model of Duchenne muscular dystrophy (DMD) shows cognitive and brain biochemical changes secondary to dystrophin deficiency in the brain, and is a relevant pre-clinical model to develop therapies for DMD.

## INTRODUCTION

Duchenne muscular dystrophy (DMD) is a fatal, X-linked genetic condition caused by dystrophin deficiency in striated muscle that affects ∼1 in 5000 male births (Mah et al., 2014). Myofibre necrosis and subsequent replacement with fibrous and adipose tissue leads to progressive muscle weakness (Sussman, 2002). Affected boys typically require a wheelchair before their teens and develop terminal cardiomyopathy and/or respiratory failure in their mid-20s (Rall and Grimm, 2012). In addition to the striated muscle features, ∼50% of DMD patients have a cognitive phenotype secondary to deficiency of dystrophin in the brain. Intellectual impairment (IQ<70) is detected in ∼30% of boys with DMD (Cotton et al., 2001), alongside a higher incidence of attention-deficit/hyperactivity disorder (ADHD) (32%), anxiety disorder (27%), autism spectrum disorders (ASD) (15%), epilepsy (6.3%) and obsessive-compulsive disorder (4.8%) (Banihani et al., 2015; Hendriksen and Vles, 2008; Pane et al., 2013). Although the therapeutic focus to date has been on correcting the muscle phenotype, encouraging progress in this area (Bengtsson et al., 2017; Goyenvalle et al., 2015; Wang et al., 2012; Amoasii et al., 2018) means that the longer-term impact of early-life cognitive deficits will likely become clearer and more pertinent in the near future, yet there have been no reported efforts to treat the brain manifestations of DMD – an urgent unmet need.

**Fig. 1.**
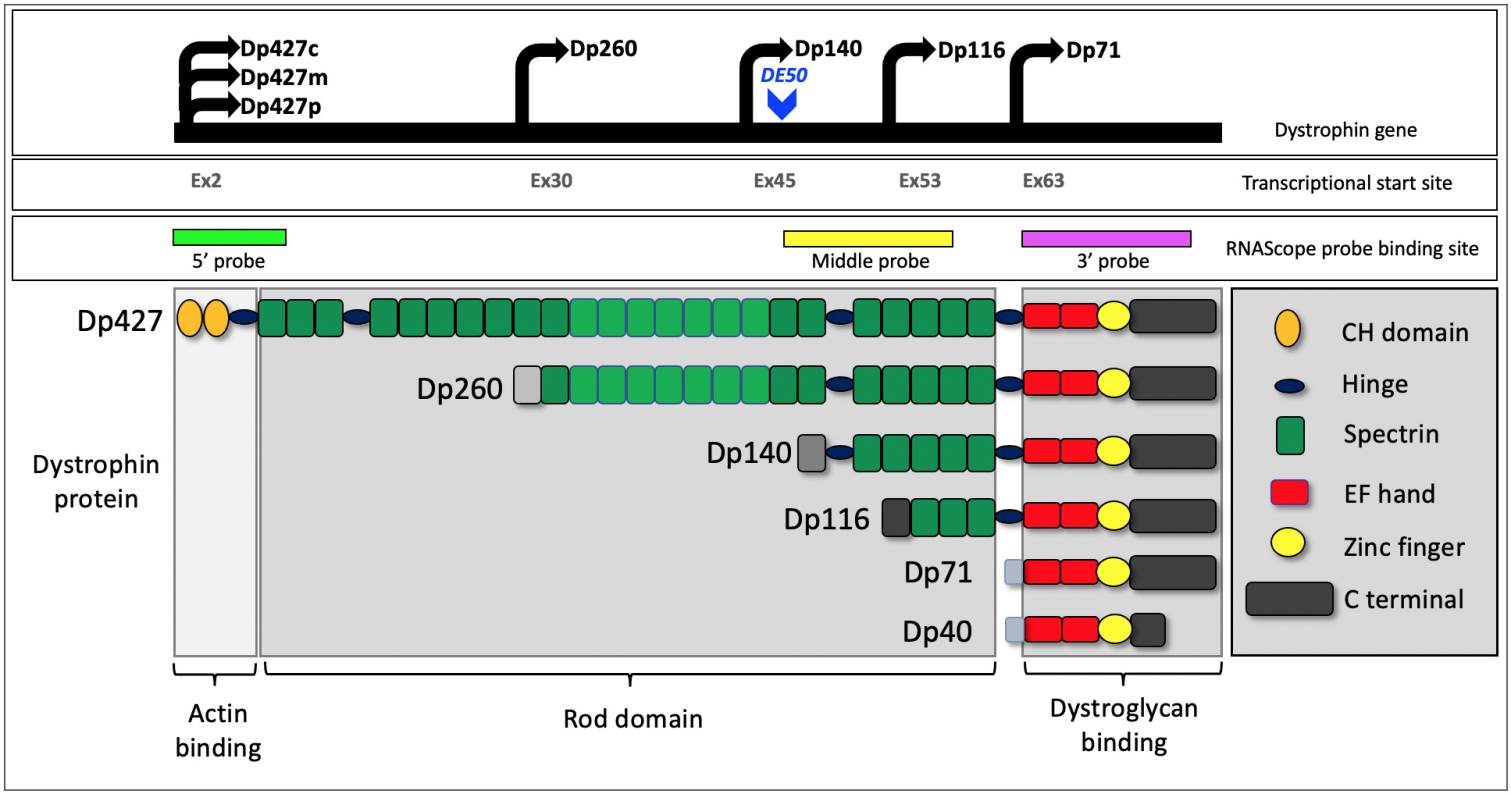
**Schematic depiction of the dystrophin gene and its transcripts.** The dystrophin gene has multiple promoters: three generating full-length isoforms (Dp427c, m, p) and four internal promoters giving rise to shorter isoforms [Dp260 (retinal isoform), Dp140, Dp116 (Schwann cell isoform) and Dp71]. A further short isoform (Dp40) is produced from alternate splicing of Dp71. Dp427 carries multiple functional domains, subsets of which are retained by the shorter isoforms. The lighter shading of spectrin repeats 11-17 indicates the additional actin-binding motif of the Dp427 and Dp260 isoforms. The site of the DE50-MD dog model mutation in exon 50 is shown, as are the target-binding sites of the RNAScope multiplex probes (5′, middle, 3′) used in this study.

The *DMD* gene contains at least seven tissue-specific promoters and two polyA-addition sites, producing several isoforms that share significant sequence conservation beyond N terminal truncations (Fig. 1). The full-length muscle isoform Dp427m has been well characterised in both its cellular localisation and function within skeletal and cardiac muscle: it bridges the myofibre cytoskeleton, maintaining a link ultimately to the extracellular matrix via the dystrophin-associated glycoprotein complex, thereby providing structural stability (Sweeney and Barton, 2000). The other dystrophin isoforms are less well characterised. In the human brain, the cortical isoform Dp427c is reported to be predominantly expressed in cortical and hippocampal (Cornu Ammonis) neurons (Nudel et al., 1989), while the Purkinje isoform Dp427p is virtually absent (Doorenweerd et al., 2017). The Dp140 isoform is robustly expressed developmentally before becoming predominantly expressed in the cerebellum postnatally (Doorenweerd et al., 2017). Finally, Dp71 has a relatively ubiquitous expression and is the most abundant dystrophin isoform in the brain (Austin et al., 1995; Lederfein et al., 1992). The function(s) of each isoform in the brain requires further investigation, but Dp427 appears to play a role in GABA-receptor clustering on postsynaptic membranes (Knuesel et al., 1999; Vaillend et al., 2010), Dp140 in early neurodevelopment (neuron differentiation and projection morphogenesis) (Doorenweerd et al., 2017) and Dp71 in aquaporin-4 and inward-rectifying potassium channel organisation (Daoud et al., 2008; Miranda et al., 2011).

Diverse mutational events within the *DMD* gene compromise dystrophin protein expression and result in the clinical manifestations of DMD. The precise location of the mutation determines which isoforms are compromised (more distal mutations have the potential to affect the expression of increasing numbers of dystrophin isoforms). Correlation of Full-Scale Intelligence Quotients (FSIQ) with the location of the *DMD* gene mutation suggests that the risk of cognitive deficits increases with cumulative loss of dystrophin isoforms. FSIQ scores are lowest in patients deficient in all dystrophin isoforms, and highest (although still below scores from individuals with intact expression) in those lacking only Dp427 (Taylor et al., 2010). Furthermore, patients lacking Dp140 have poorer performance in neuropsychological testing, including verbal and visual memory, attention and executive function (Bardoni et al., 2000; Chamova et al., 2013; Taylor et al., 2010), alongside more severe loss of grey matter and reduction in total brain volume (Doorenweerd et al., 2014), compared with patients with intact Dp140 expression. Given the suspected predominantly neurodevelopmental role of Dp140, the consequences of its deficiency might be established at or before birth, and postnatal restoration might concomitantly offer little therapeutic benefit. In contrast, if an important functional role of Dp427 is confirmed postnatally and into adulthood, initial therapeutic strategies targeting this isoform might be more feasible and effective.

Much of the available information on dystrophin expression and function is derived from animal models, including mouse (Bulfield et al., 1984), rat (Larcher et al., 2014), dog (Walmsley et al., 2010; Kornegay, 2017) and pig (Klymiuk et al., 2013). Dogs are a particularly useful model as their severity and progression of muscle disease more closely mimics that of humans, compared with the relatively mild phenotype of commonly used murine models (McGreevy et al., 2015). Cognitive and neurobehavioural dysfunction are recognised clinical entities in dogs, and their detailed characterisation is feasible because of the relative ease with which dogs can be trained (Packer et al., 2018a,b; Schütt et al., 2016). Furthermore, a recent study of dystrophin-deficient muscular dystrophy in miniature poodles harbouring a large deletion on the X chromosome (with loss of the entire *DMD* gene) reported learning difficulties and episodes of abnormal behaviour potentially compatible with a neuropsychiatric syndrome caused by brain dystrophin deficiency (Sanchez et al., 2018). Interestingly, although mouse models of DMD show a range of deficits on cognitive testing, differences have been reported in dystrophin isoform expression between human and murine brain (particularly in expression of the Purkinje isoform of Dp427p) (Doorenweerd et al., 2017). Finally, dogs are more comparable in size and brain structure (e.g. convoluted cerebral cortex) to humans than rodents, so facilitating the development of drug administration techniques, while their longer lifespan enables assessment of long-term efficacy and tolerability of treatments. The unique DE50-MD dog model carries a missense mutation in the donor splice site of exon 50 (resulting in exclusion of exon 50 from the mature mRNA) (Walmsley et al., 2010), a locus that falls within the major human DMD mutational hotspot (exons 45-53). In contrast, the Golden Retriever dog model has a mutation in intron 6 (Koenig et al., 1989; Sharp et al., 1992), far from this hotspot. The mutation site makes the DE50-MD model particularly attractive for demonstration of efficacy of certain molecular therapies, as therapeutic skipping of exon 51 can restore the Dp427 reading frame. Recently, we reported the first successful use of CRISPR/Cas9-mediated gene editing to elicit reframing in muscle of the DE50-MD dog (Amoasii et al., 2018).

In this study, we show that the DE50-MD canine model of DMD has a detectable cognitive and neurobehavioural phenotype. We evaluate dystrophin expression in the normal adult canine brain and confirm expression of Dp427, Dp140 and Dp71, with regional expression patterns that reflect those of the human brain. We demonstrate that the DE50-MD dog is deficient in Dp427 protein expression but retains expression of Dp140 and Dp71. Together, our results show that the DE50-MD dog is a translationally relevant pre-clinical model with which to study brain dystrophin function and to develop therapeutic strategies for the neurological sequelae of DMD.

## RESULTS

### Cognitive testing

To identify subtle cognitive and behavioural deficits while attempting to draw parallels with the cognitive phenotype recognised in boys with DMD as far as feasible, a broad battery of tests was utilised. Novel skill acquisition and perseverative responding, exploration behaviour and response to novelty, attention, motivation, problem solving and blink rate were evaluated. Additionally, perseveration in extinction has been linked with autism (Hughes et al., 1994; Landry and Mitchell, 2021; Pascualvaca et al., 1998) and a reduced blink rate with ADHD (Konrad et al., 2003), thus facilitating assessment of potentially associated neurobehavioural disorders.

#### Novel olfactory cue

Response to novelty was assessed with olfactory cues [*n*=8 DE50-MD, *n*=6 wild type (WT), Mann–Whitney test]. DE50-MD dogs spent less time investigating the olfactory cue [DE50-MD, 18 s (range 4-27 s); WT, 24.5 s (22-65 s); U=8, *P*=0.039], with fewer returns to the cue compared with WT dogs [DE50-MD, 1 (1-2); WT, 2 (2-4); U=2, *P*=0.002]. No significant difference was found in time to detect the novel cue [DE50-MD, 6 s (1-35 s); WT, 2.5 s (2-20 s); U=15.5, *P*=0.297], nor number of markings of the cue [DE50-MD, 1 (0-1); WT, 1 (0-2); U=14, *P*=0.224].

#### Novel object

**Fig. 2.**
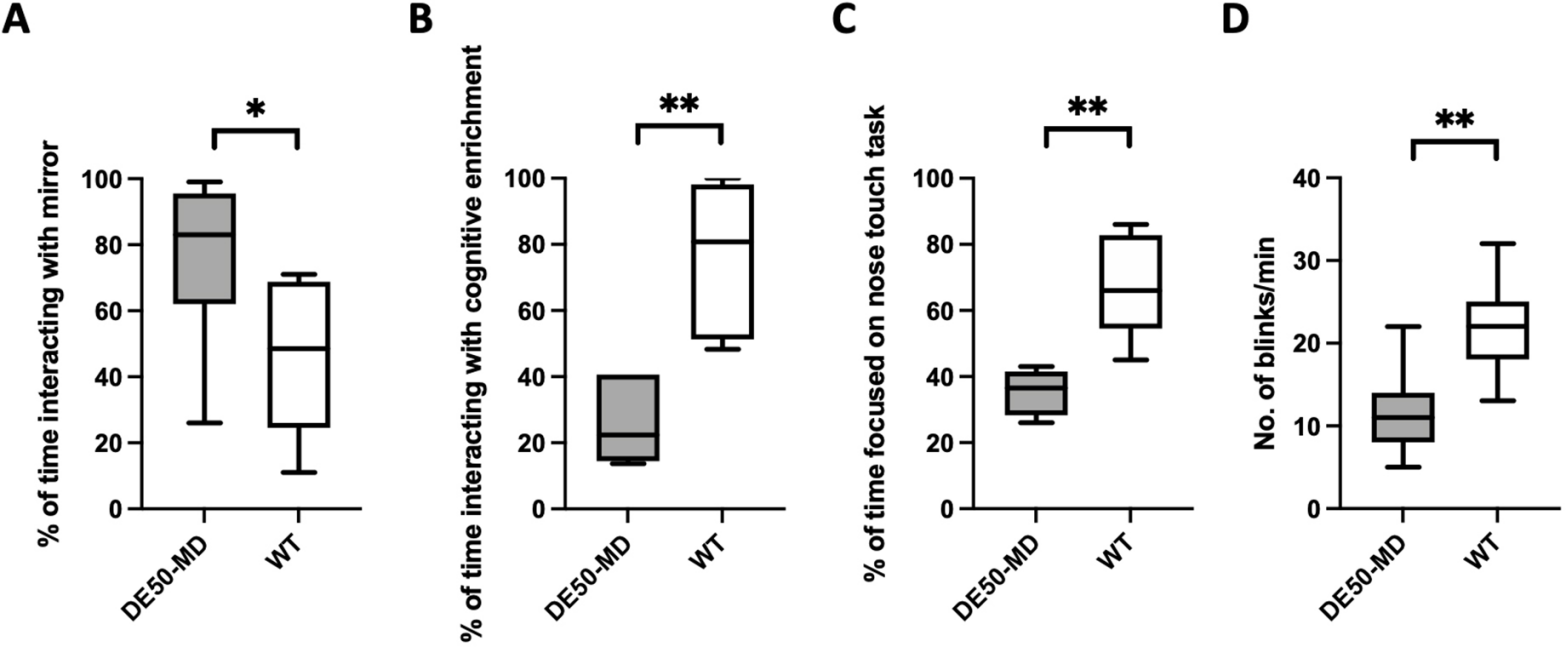
**Behavioural testing reveals differences in response to novelty, attention and blink rate in DE50-MD dogs compared with age-matched WT controls.** (A) DE50-MD dogs showed increased duration of interaction with a novel object (mirror) compared with WT controls. (B) DE50-MD dogs showed reduced duration of focused interaction with a cognitive enrichment puzzle toy. (C) Duration of focused attention was reduced in DE50-MD dogs when assessed for acquisition and extinction of a nose touch task. (D) DE50-MD dogs have a reduced blink rate compared with WT controls (*n*=6-8 DE50-MD, *n*=6-8 WT; *P<0.05, **P<0.01, Mann–Whitney test).

Response to placing a mirror at the kennel door was recorded (*n*=8 DE50-MD, *n*=8 WT, Mann–Whitney test). DE50-MD dogs spent less time barking at the mirror [DE50-MD, median 0 s (range 0-50 s); WT, 55 s (0-215 s); U=7, *P*=0.006] and returned to investigate the mirror less often than WT dogs [DE50-MD, 7 (1-17); WT, 17 (3-28); U=9, *P*=0.007]. However, they showed a greater overall proportion of time interacting with the mirror [DE50-MD, 83% (26-99%); WT, 49% (11-71%); U=12, *P*=0.021) (Fig. 2A). All WT dogs jumped up at the kennel bars in response to the mirror, while none of the DE50-MD dogs did, likely reflecting muscle weakness.

#### Response to a cognitive enrichment

Ability to obtain food treats from a puzzle toy was assessed to evaluate problem-solving skills and motivation (Table 1, Fig. 2B). No difference was detected in time to detect the cognitive enrichment between DE50-MD and WT dogs. All DE50-MD dogs failed to obtain a treat reward after 2.5 min of recording; hence, one plastic puzzle piece was removed to reveal the underlying treat. One DE50-MD dog then obtained a single treat without assistance, and the remaining five failed to obtain any treats. When shown a treat within the puzzle, all DE50-MD dogs immediately ate the treat, suggesting that inappetence or disinterest in the specific treat offered did not influence performance. All control dogs obtained treats without assistance; five completed the puzzle (i.e. obtained all nine treats) in a mean time of 130 s, and the remaining control dog obtained 8/9 of the available treats.

**Table 1.**
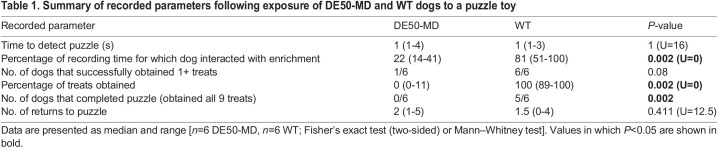
Summary of recorded parameters following exposure of DE50-MD and WT dogs to a puzzle toy

#### Acquisition and extinction learning (nose touch response)

Ability to learn a novel task, and subsequent reversal of that learning were evaluated [*n*=6 DE50-MD, *n*=6 WT; Mann-Whitney test or Fisher’s exact test (two-sided)]. No significant difference was found in time taken to first successful nose touch between DE50-MD and WT dogs [DE50-MD, median 44 s (range 26-82 s); WT, 65 s (53-88 s); U=6, *P*=0.065]. The numbers of behavioural changes observed during the training period (e.g. from focused on the task to exploring the room) were not significantly different between DE50-MD and WT dogs [DE50-MD, median 4 (3-9); WT, 6 (1-15); U=9.5, *P*=0.197]. However, DE50-MD dogs spent significantly less time focused on the task, compared with WT dogs [DE50-MD, 37% (36-43%); WT, 64% (45-86%), U=0, *P*=0.004] (Fig. 2C). Furthermore, 6/6 DE50-MD dogs were excluded as non-responders and so did not reach the extinction phase, compared with 2/6 WT dogs (*P*=0.06).

#### Spontaneous eye blink rate

Median eye blink rate (EBR) was lower in DE50-MD dogs compared with WT littermates [DE50-MD, 11 (5-22); WT, 22 (13-32); U=15, *P*=0.001] (*n*=8 DE50-MD, *n*=6 WT) (Fig. 2D). Repetitive stimulation of the medial canthus over 60 s to elicit the palpebral reflex resulted in a consistent and complete blink with full eyelid closure and no evidence of fatigue in both DE50-MD and WT dogs; hence, facial paresis as a result of the muscle pathology does not appear to contribute to the reduced EBR in DE50-MD dogs.

### Dystrophin isoforms in the brain: protein

**Fig. 3.**
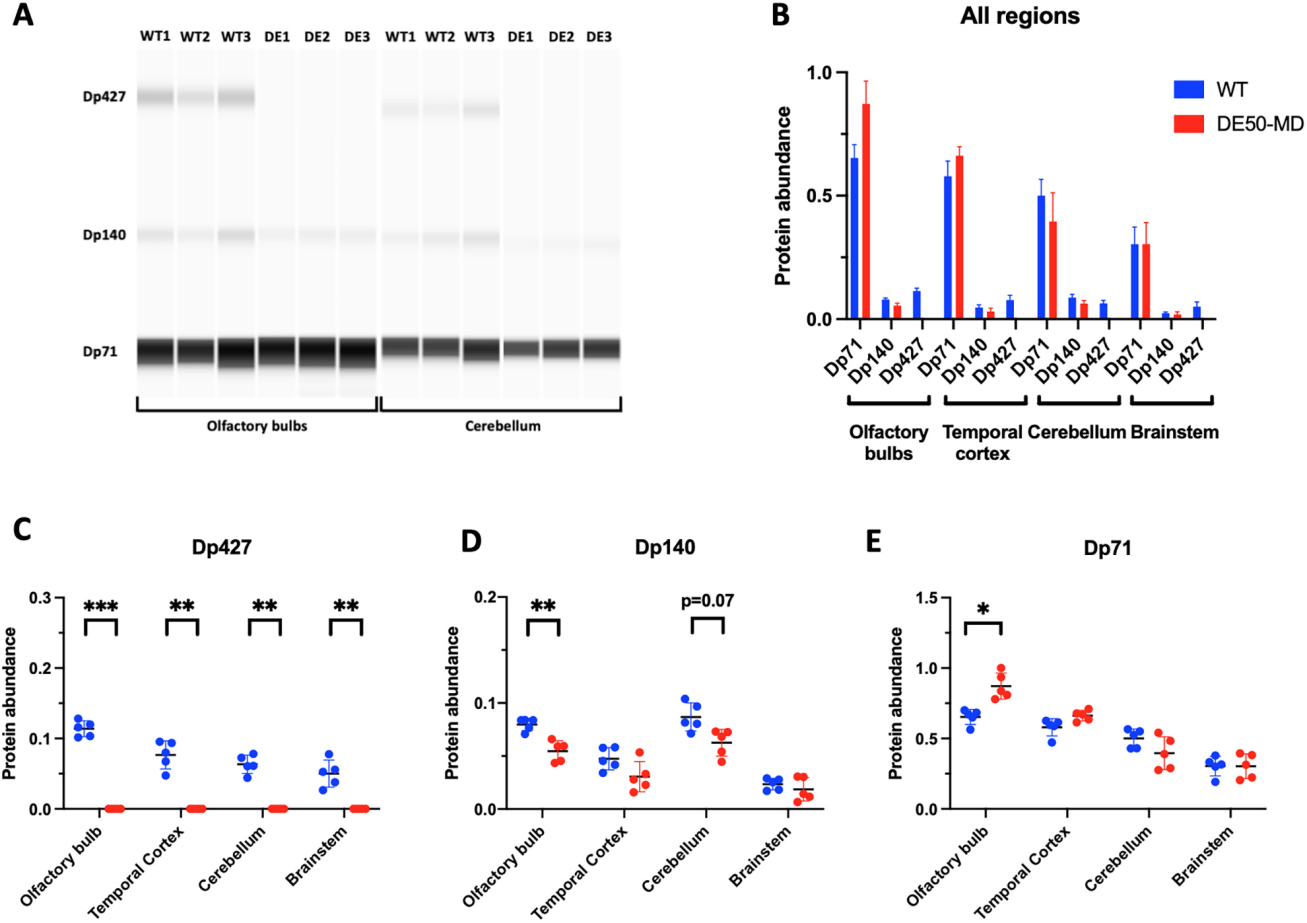
**Capillary immunoelectrophoresis protein analysis detects dystrophin protein (Dp427, Dp140 and Dp71) in the canine olfactory bulbs, cerebral cortex (temporal), cerebellum and brainstem.** (A) Three dystrophin isoforms are detected in the canine WT brain: Dp427, Dp140 and Dp71. DE50-MD dogs express Dp140 and Dp71, but not Dp427. (B) Dp71 is the most abundant dystrophin isoform in the canine brain. Data are normalised to total protein expression and shown as relative abundance. (C) Dp427 is not detected in the DE50-MD dog brain. (D) Dp140 expression is decreased in the olfactory bulb and cerebellum of DE50-MD dogs. (E) Dp71 expression is higher in the olfactory bulbs in DE50-MD dogs compared with WT. In B, data are shown as mean±s.d.; in C-E, data are shown as individual animal samples, mean±s.d. (*n*=5 DE50-MD, *n*=5 WT; *P<0.05, **P<0.01, ***P<0.001, one-way ANOVA with Sidak's multiple comparisons test).

Protein immunoelectrophoresis confirmed expression of three dystrophin isoforms within the WT canine brain: full length Dp427, alongside two shorter isoforms, Dp140 and Dp71 (Fig. 3; Fig. S1). All three isoforms were detected in all studied regions of the brain (olfactory bulbs, cerebral cortex, cerebellum and brainstem). As expected, Dp71 was the most abundant isoform, with robust expression in all studied regions, while Dp427 and Dp140 were expressed at lower levels. The retinal (Dp260) and peripheral nerve (Dp116) isoforms were not detected in the canine brain.

In DE50-MD dogs, Dp427 protein was not detected in any of the studied brain regions, but both Dp140 and Dp71 were expressed. Levels of these latter isoforms broadly mirrored the pattern found in the WT brain, Dp140 expression exhibited a modest but consistent reduction, particularly in the olfactory bulbs (Fig. 3D), and Dp71 expression was found to be higher in the olfactory bulbs of DE50-MD dogs (Fig. 3E).

**Fig. 4.**
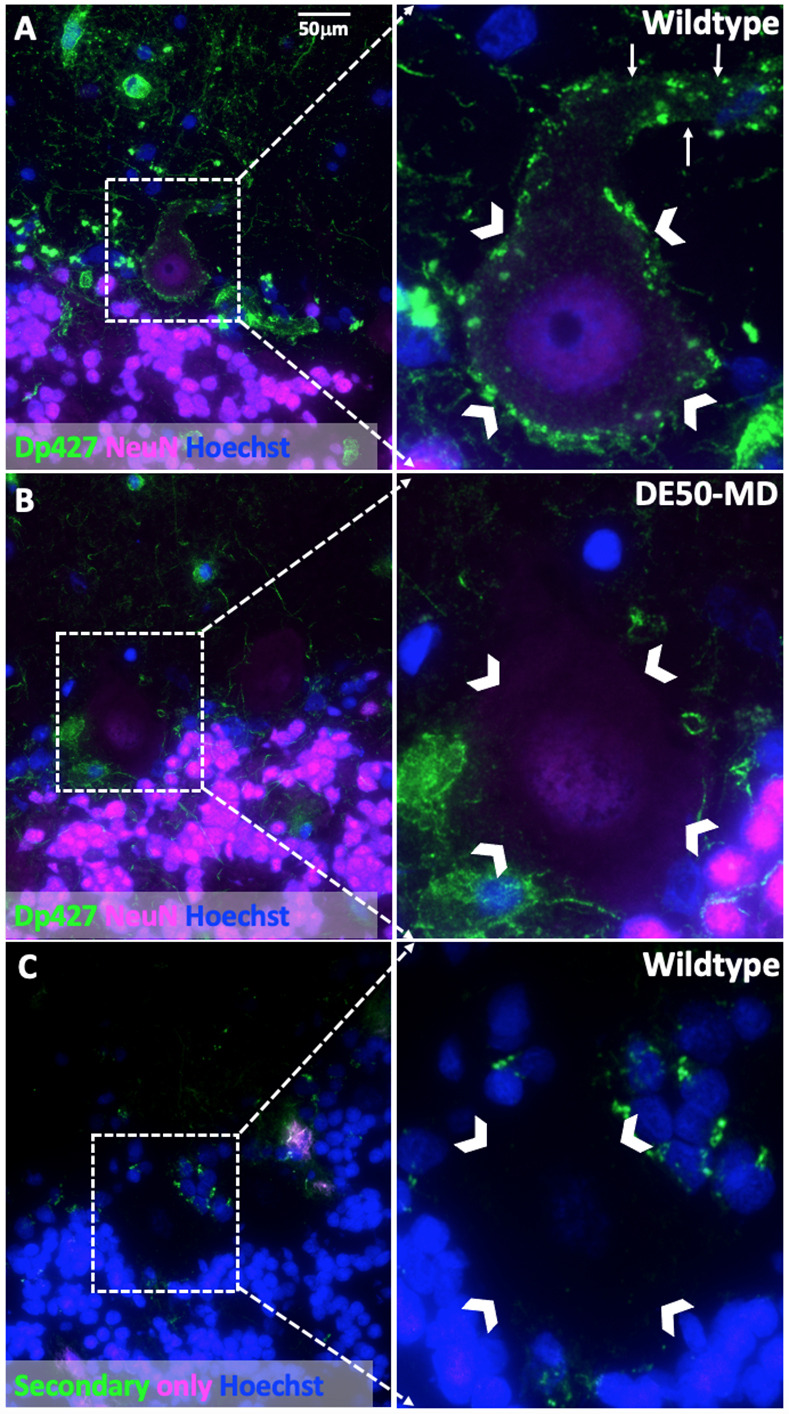
**Immunolabelling of dystrophin in the canine cerebellar cortex with co-labelling for NeuN as a neuronal marker.** (A) Cerebellar cortex of a WT dog: peripheral punctate staining of dystrophin (Dp427) in the Purkinje neuron cell body (carets) and apical dendrite (arrows) is seen. (B) Cerebellar cortex of a DE50-MD dog: no dystrophin-positive staining of the Purkinje neuron is visible (carets delineate Purkinje neuron cell body). [Dystrophin detected with ManDys8 antibody, which detects Dp427 and Dp260 (not expressed in brain)]. (C) WT canine cerebellum immunolabelled with secondary antibodies only (negative control) plus Hoechst. No primary antibodies were applied to evaluate background fluorescence and non-specific labelling by secondary antibodies. Foci of fluorescence are detected in all three layers of the cerebellar cortex, but no perineuronal staining is detected (carets delineate Purkinje neuron cell body).

**Fig. 5.**
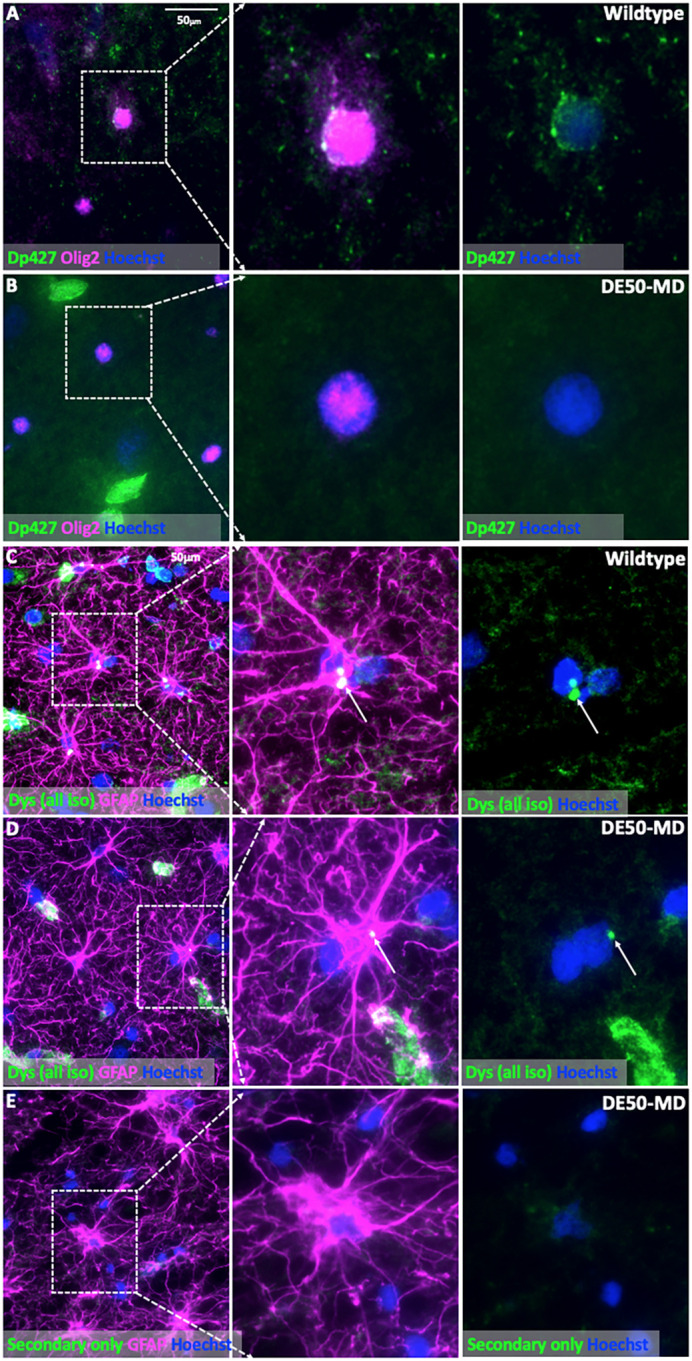
**Immunolabelling of dystrophin in the canine brain with co-labelling for Olig2 as a marker of oligodendrocytes or GFAP as a marker of astrocytes.** (A) Cerebellar cortex from a WT dog: infrequent Olig2^+^ oligodendrocytes show subtle punctate perinuclear dystrophin-positive staining. (B) Cerebellar cortex from a DE50-MD dog: no dystrophin-positive staining of Olig2^+^ oligodendrocytes was detected in DE50-MD dogs. (C,D) Occipital cortex of a WT dog (C) and a DE50-MD dog (D): some GFAP^+^ astrocytes show small foci of dystrophin-positive perinuclear staining (arrows). (E) Occipital cortex of a DE50-MD dog: no anti-dystrophin antibody was applied (negative control). Green staining represents non-specific background staining from the secondary antibody. Faint green background staining is visualised but no discrete perinuclear foci as seen in C and D. In A and B, dystrophin was detected with ManDys8 antibody, which detects Dp427 and Dp260 (not expressed in brain); in C and D, dystrophin was detected with the Abcam15277 antibody, which detects all dystrophin isoforms. ManDys8 staining was not possible due to species cross-reactivity with the anti-GFAP antibody found to reliably label canine astrocytes (both raised in mouse).

A panel of antibodies was tested in WT canine muscle and brain (Table S1); although all detected dystrophin in skeletal muscle samples (Fig. S2), only three of the tested antibodies consistently revealed dystrophin-positive staining in the brain [ManDys8 (detects Dp427 and Dp260), ManEx59B (detects Dp427, Dp260 and Dp140) and Abcam15277 (detects all isoforms)]. These antibodies revealed peripheral, punctate dystrophin-positive staining of neuronal cell bodies. Purkinje neurons of the cerebellum showed dystrophin-positive punctate staining extending around the periphery of the cell body and along the apical dendrite (Fig. 4A; Fig. S3). No positive staining of the granular neurons of the cerebellum was detected with immunolabelling. Cerebral cortical neurons also showed dystrophin-positive punctate staining of the cell body (Figs S3 and S4). A secondary antibody-only control (no anti-dystrophin anitbody applied) showed no comparable punctate staining, suggesting that this staining is specific for dystrophin (Fig. 4C). Sporadic (median 14%) Olig2^+^ cells (oligodendrocytes) showed a thin rim of peripheral staining around the cell body (Fig. 5A; Fig. S3). Dystrophin-positive staining in GFAP^+^ cells (astrocytes) showed a different pattern, with small, dense perinuclear foci detected in a median of 38% of cells (Fig. 5C; Fig. S3). A secondary antibody-only control showed no similar foci (Fig. 5E).

In DE50-MD dog brains, no perineuronal dystrophin-positive staining was detected, consistent with the absence of Dp427 (Fig. 4B; Figs S3 and S4). The peripheral dystrophin-positive staining detected in WT Olig2^+^ cells was not detected in the DE50-MD dogs, supporting the expression of Dp427 by oligodendrocyte lineage cells (Fig. 5B). A small perinuclear focus of dystrophin-positive staining was detected in occasional (median 8%) GFAP^+^ cells, suggesting that astrocytes express Dp140 and/or Dp71 (Fig. 5D).

### Dystrophin isoforms in the brain: mRNA

**Fig. 6.**
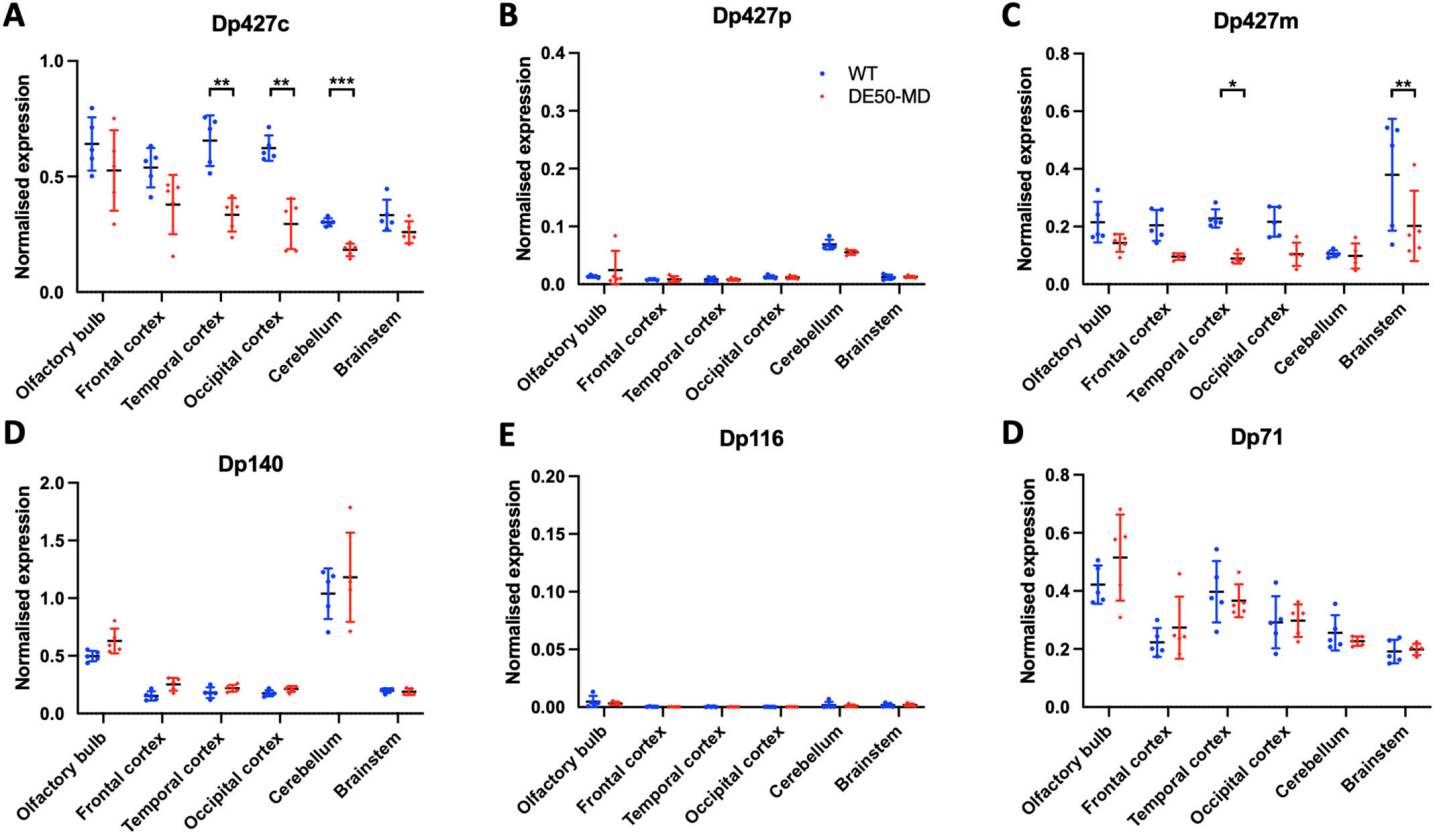
**Relative expression of Dp427, Dp140 and Dp71 isoform mRNA assessed by RT-qPCR in six regions of the canine brain.** Data are converted to relative quantities and normalised to the geometric mean of three previously validated reference genes (*SDHA*, *UBC* and *YWHAZ*) (Crawford et al., 2021). Full-length dystrophin (Dp427) can be transcribed from three discrete promoters, giving rise to muscle (Dp427m), cortical (Dp427c) and Purkinje (Dp427p) isoforms. Further internal promoters give rise to shorter isoforms, including Dp140, Dp116 and Dp71. (A) Dp427c mRNA is detected in all six studied regions of the canine brain, with significantly reduced expression in DE50-MD dogs compared with WT in the cerebral cortex (temporal and occipital) and cerebellum. (B) Dp427p mRNA is detected at low levels in the cerebellum. (C) Dp427m mRNA is expressed in all six regions, with significantly lower expression in DE50-MD dogs in the temporal cortex and brainstem. (D) Dp140 mRNA is expressed in all six regions but is most abundant in the cerebellum. No difference is detected in expression between DE50-MD and WT dogs. (E) Dp116 is not detected in any of the studied brain regions. (F) Dp71 is expressed in all six regions. Again, there is no detected difference in expression between DE50-MD and WT dogs. Data are shown as individual animal samples, mean±s.d. (*n*=5 DE50-MD, *n*=5 WT; *P<0.05, **P<0.01, ***P<0.001, one-way ANOVA with Sidak's multiple comparisons test).

Reverse transcription quantitative real-time PCR (RT-qPCR) using primers to the unique first exon of each dystrophin isoform was employed to detect and quantify mRNA from three regions of the cerebral cortex (frontal, temporal and occipital), the olfactory bulbs, cerebellum and brainstem (Fig. 6). All three isoforms of Dp427 (c, m and p) were detected. Dp427c mRNA was the most abundant, found in each of the six studied brain regions. Levels of Dp427c were ∼2-fold lower in the DE50-MD cerebral cortex (temporal and occipital) and cerebellum compared with WT. Dp427m expression was modest compared with Dp427c, but this isoform was also present in all regions studied, with reduced levels again detected in DE50-MD samples (particularly temporal cortex and brainstem). Expression of Dp427p was very low; this isoform was detected in the cerebellum but levels were orders of magnitude lower than for Dp427c or Dp427m.

Dp140 mRNA was present in all studied regions but was most abundant in the cerebellum. Dp71 mRNA was ubiquitously expressed, although transcript levels for this isoform appeared comparable to those for Dp427m and Dp427c. No significant differences were found in normalised expression of Dp140 and Dp71 between WT and DE50-MD dogs.

To detect dystrophin mRNA molecules at the subcellular histological level, we employed a multiplex RNAScope *in situ* hybridisation (ISH) approach as described previously (McGreevy et al., 2015). Three probe sets were used to recognise the 5′, middle and 3′ regions of the Dp427 transcript: Dp427 transcripts label with all three probes (hence three distinct fluorophores), Dp140 transcripts with middle and 3′ probes, while Dp71 mRNA binds 3′ probe alone. Typically, RNAScope ISH produces small, punctate fluorescent foci, each corresponding to a single transcript molecule. Given the size of the dystrophin gene [transcription of full-length Dp427 mRNAs takes ∼16 h, and the mRNAs are co-transcriptionally spliced (Tennyson et al., 1995)], nuclei may contain multiple nascent (incomplete) transcripts at the dystrophin gene locus. These nascent mRNAs will be recognised by this ISH method, producing large nuclear fluorophore deposits of 5′ and middle probe (Dp427), or of middle probe alone (Dp140). Dp71 mRNAs are estimated to require only ∼1 h for transcription and will thus behave more conventionally, as will mature mRNAs of all isoforms (visualised as small foci of the appropriate probes).

**Fig. 7.**
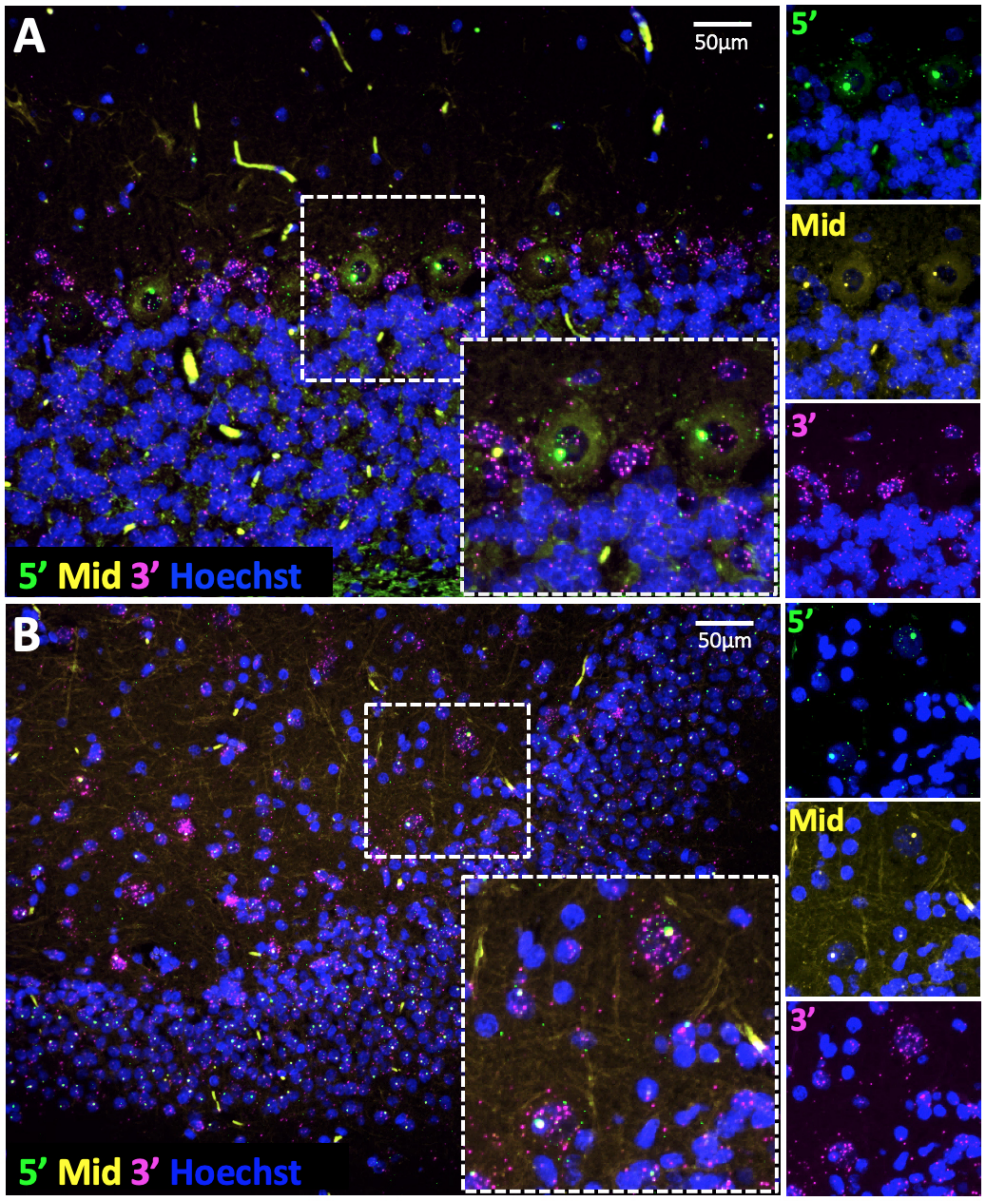
**RNAScope *in situ* hybridisation reveals dystrophin mRNA expression in the DE50-MD cerebellar cortex and dentate gyrus of the hippocampus.** Serial oligonucleotide probes designed to recognise the 5′ (green), mid-transcript (yellow) and 3′ (magenta) regions of dystrophin mRNA enabled transcript detection at subcellular levels. (A) Binding of all three probes was detected in the Purkinje neurons of the cerebellar cortex, consistent with Dp427 expression. Mid and 3′ probe binding was detected at low levels in the granular neurons, consistent with Dp140 expression. (B) Hippocampal neurons of the dentate gyrus showed binding of all three probes, with large foci of 5′ binding at the dystrophin locus, consistent with Dp427 expression.

In both DE50-MD and WT dogs (*n*=2 per genotype), large foci of 5′ probe binding were detected in the nucleus of neurons throughout the cerebral cortex, hippocampus, cerebellum and brainstem, each with a smaller co-localising focus of mid-transcript probe binding and surrounding punctate foci of all three probes, consistent with Dp427 expression (Fig. 7; Fig. S5). Positive and negative controls are shown in Fig. S6. Intense labelling of nascent Dp427 appeared comparable between genotypes; quantification of punctae was not performed as samples were only available for two WT dogs. The 3′ and mid-transcript probe signal in the absence of 5′ probe is consistent with Dp140 expression and was detected at low levels in the granular neurons of the cerebellar cortex (Fig. 7A) and in ependymal cells (Fig. S5B). This contrasts with the immunolabelling analysis, with which we were unable to detect dystrophin expression in the cerebellar granular neurons, and likely reflects the greater sensitivity of RNAScope ISH, which can discern single dystrophin transcripts. Foci of 3′ probe alone (consistent with Dp71 expression) were evident in the nuclei of ependymal cells and modified ependymal cells of the choroid plexus (Fig. S5B). Infrequent glia showed binding of all three probes, validating the immunolabelling and confirming that dystrophin is expressed by glia in the canine brain (Fig. S5C).

Thus, these techniques, in combination, confirm that dystrophin is extensively expressed in the canine brain. A high-sensitivity immunoelectrophoresis approach confirmed expression of full-length dystrophin as well as two shorter isoforms, Dp140 and Dp71. Immunolabelling revealed that Dp427 is expressed in small punctae around the periphery of neuronal cell bodies throughout the brain but was inadequately sensitive to reliably detect the shorter isoforms. RT-qPCR enabled isoform discrimination and confirmed expression of Dp427c, Dp427m, Dp140 (most abundant in the cerebellum) and Dp71 mRNA throughout the canine brain, with low levels of Dp427p in the cerebellum. Finally, a high-sensitivity ISH technique confirmed widespread Dp427 expression by neurons throughout all studied regions of the canine brain. Furthermore, it confirmed Dp427 expression by glial cells, Dp140 expression by granular neurons of the cerebellum and Dp71 expression by ependymal cells. Immunoelectrophoresis and immunolabelling confirmed that DE50-MD dogs do not express Dp427 protein and show a reduction in Dp140 expression, while RT-qPCR revealed reduced Dp427m and Dp427c transcripts compared with WT.

## DISCUSSION

Development of therapeutic strategies to correct the neurobehavioural and cognitive deficits associated with DMD will require a detailed understanding of the precise functions and regional expression patterns of dystrophin isoforms within the brain. Canine models of DMD have been pivotal in the continuing development of muscle-directed therapeutic strategies for DMD (Amoasii et al., 2018; Kodippili et al., 2018; Song et al., 2019). However, to date, there have been no studies investigating dystrophin expression in the canine brain, nor the consequences of its deficiency. In this study, we have utilised a range of techniques to detect dystrophin protein and mRNA within the canine brain and show that dystrophin isoform expression is comparable to that of the human brain yet differs from that of the mouse brain (in which Dp427p is more abundantly expressed). Furthermore, we have identified a cognitive phenotype in dogs deficient in Dp427.

We hypothesised that dogs with DMD, like their human counterparts, have cognitive and neurobehavioural deficits as a result of brain dystrophin deficiency. In boys with DMD, difficulties with verbal memory, reading, syntax comprehension and story recall, alongside a lower FSIQ, have been documented (Nicholson et al., 1993; Taylor et al., 2010; Cotton et al., 2001), parameters that are either not applicable or not possible to assess in canine models. Thus, to study cognitive function more broadly in our canine model, we utilised tests that evaluated novel skill acquisition and perseverative responding, exploration behaviour and response to novelty, attention, motivation, problem solving and blink rate. Unlike the DE50-MD dogs, in which muscle paresis is typically evident and progressive from ∼1-2 months of age, *mdx* mice (a commonly used model of DMD with a spontaneous mutation in exon 23) do not display motor disabilities until at least 6 months of age, allowing for detailed behavioural testing in the absence of confounding motor defects (Remmelink et al., 2016; Muntoni et al., 1993). Reported findings include deficits in passive avoidance behaviour (Muntoni et al., 1991), enhanced defensive freezing (Sekiguchi et al., 2009), long delays in spontaneous alternation tasks (Vaillend et al., 1995), and impaired long-term spatial and recognition memory (Vaillend et al., 2004). A requirement of our battery of tests was to assess cognitive function independently of the marked physical impairments of the canine muscle pathology. Tests were specifically chosen to be of short duration, with no requirement for significant motor activity nor physical exertion. We additionally selected tests that did not require prolonged training periods to allow direct application in future clinical trials in which such training is not feasible. Despite this, we cannot totally exclude that the muscle pathology and paretic phenotype of DE50-MD dogs did not influence our test results, or contribute to a lack of motivation to interact and thus to an apparent reduction in focus.

Declining response to novelty has been detected with ageing (Daffner et al., 1994) and Alzheimer's disease in humans (Daffner et al., 1999). Although both WT and DE50-MD dogs detected novel cues, DE50-MD dogs showed reduced interaction with a novel olfactory cue, spending less time investigating the object and returning to explore the object less frequently than WT dogs. Furthermore, in response to a novel cognitive enrichment, specifically a puzzle toy, DE50-MD dogs failed to retrieve the food rewards located inside the toy. In contrast to a nose touch task in which there was interaction with the investigator and the food reward was directly accessible to the dogs, the puzzle toy required independent exploration and self-driven problem solving to locate the food rewards. Thus, dystrophin deficiency in the canine brain may reduce exploratory behaviour, motivation to explore novel cues and problem-solving skills. In the *mdx* mouse, intact novelty-seeking behaviour has been reported (Vaillend et al., 2004), but enhanced fearfulness was associated with a reduced exploration of novel objects when assessed in an open field test (Vaillend and Chaussenot, 2017). Enhanced fearfulness was not specifically assessed in the DE50-MD dogs, but might contribute to their reduction in exploration behaviours. Furthermore, the shape, size and novel visual appearance of the plastic ball used in the olfactory test might have induced fear or anxiety. The robust expression of Dp427 in the olfactory bulbs implies that DE50-MD dogs might exhibit an olfactory deficit; however, DE50-MD animals showed no delay in time to detect the cue compared with WT controls. Given the greater olfactory acuity of dogs compared with humans, the olfactory test we employed might be insufficiently sensitive to detect subtle olfactory deficits.

When exposed to a mirror, the DE50-MD dogs spent longer engaging with it than did the WT dogs. The mirror test was originally developed to assess self-recognition in primates (Gallup, 1968) and has been used in carnivorous species to evaluate social motivation (Noer et al., 2015). In a study by Siwak et al. (2001), cognitively impaired dogs showed reduced exploration of novel objects and people, but a prolonged interaction with a mirror compared with age-matched dogs without cognitive impairment. The authors discuss that a disruption of frontal lobe circuitry could account for the reduced responsiveness to novel stimuli, as well as failure to habituate to a mirror reflection.

DE50-MD dogs showed a comparable rate of learning of a new task (nose touch to receive a food reward) to that of WT littermate controls. In agreement, normal learning of a new task, including a bar-pressing task and navigation of a water maze, have been demonstrated in the *mdx* mouse (Vaillend et al., 2004, 1995). Interestingly, however, DE50-MD dogs showed a reduction in the percentage of the training session for which they remained focused on the task, and 0/6 DE50-MD dogs reached the extinction phase of the exercise. This may suggest that brain dystrophin deficiency compromises ability to maintain focused attention and engagement with a task. One of the more consistent and well-documented effects of prefrontal cortex damage in humans is perseveration, in which patients are unable to switch their behavioural choices when the situation or environment changes (Luria, 1969; Milner, 1964). Thus, assessment of perseverative errors in the extinction phase of the nose touch task could have provided an indication of prefrontal cortex function in the dystrophin deficient brain. However, as no DE50-MD dogs progressed to the extinction phase of the test, we were unable to evaluate this.

EBR has been used as an indicator of dopaminergic function, with early studies in primates showing a reduction in EBR with destruction or inhibition of dopaminergic neurons (Lawrence et al., 1991; Mavridis et al., 1991). Studies of human patients with Parkinson's disease, a condition characterised by severe progressive loss of dopaminergic neurons in the striatum, identified a reduced EBR (Agostino et al., 2008; Bologna et al., 2009; Fitzpatrick et al., 2012). The precise neural circuitry through which dopamine modulates EBR, as well as the potential influence of other signalling mechanisms, remains to be fully characterised. The DE50-MD dogs had a lower EBR compared with that of WT controls, potentially consistent with a reduction in dopaminergic signalling. Fatigue was not detected on repetitive stimulation of the palpebral reflex, suggesting that facial paresis secondary to muscle pathology does not significantly contribute to reduced EBR in DE50-MD dogs. Further studies are needed to investigate dopaminergic function in the DE50-MD dog.

Notwithstanding the possible interaction of musculoskeletal effects, our data provide compelling evidence of a neurocognitive phenotype in DE50-MD dogs. Selective mitigation of the musculoskeletal or brain phenotype with targeted (tissue-regional) treatments might, in future, be a suitable way to confirm the suspected primary brain phenotype in these animals. Furthermore, comparison to other canine models of DMD may reveal important mutation-specific differences in the resultant cognitive phenotype.

All dystrophin isoform transcripts carry unique first exons (with the exception of Dp40), but otherwise share identical dystrophin sequence, resulting in very high identity at both nucleotide and protein level. Western blotting and immunoelectrophoresis can identify isoform proteins by virtue of size, and PCR targeted to unique first exon sequence can distinguish isoforms at mRNA level, but these approaches typically use tissue homogenates and so lose spatial information. Thus, to provide spatial information, we used immunolabelling and RNAScope ISH. Histological detection of dystrophin protein can be limited by the typically low levels of protein, close to or below the limit of detection, and, indeed, we were unable to discern the smaller dystrophin isoforms by immunolabelling. RNAScope ISH offers extremely high specificity (target sequences of ∼1000 bases) and sensitivity, with single mRNA transcripts resolved as punctate fluorescent foci with high signal to noise ratio (Hildyard et al., 2020a,b). Furthermore, its capacity for multiplexing enables simultaneous detection of multiple target mRNA species each labelled with a distinct fluorophore. In this and a previous study (Hildyard et al., 2020a), we multiplex within a single transcript using probes targeted to the 5′, middle and 3′ regions of dystrophin mRNA, and are able to detect individual isoform transcripts with single-cell resolution. By using a combination of these complementary techniques, we have characterised dystrophin isoform protein and mRNA expression within the adult canine brain.

Protein immunoelectrophoresis confirmed that full-length dystrophin protein is expressed throughout the WT canine brain along with two shorter isoforms, Dp71 and Dp140. These three isoforms are also found in mouse and human brain (Bar et al., 1990; Lidov et al., 1995; Górecki et al., 1992). Dp260 (the retinal isoform) (D'Souza et al., 1995) and Dp116 (the peripheral nerve isoform) (Byers et al., 1993) were not detected in canine brain.

Immunolabelling for dystrophin revealed punctate perineuronal staining throughout the brain, most clearly discerned in the cerebellar Purkinje and cortical pyramidal neurons. Comparable punctate dystrophin-positive staining of soma and dendrites of Purkinje neurons of the cerebellum and of pyramidal neurons of the cerebral cortex has been reported in WT mice but is absent in *mdx* mice that are deficient in Dp427 (Lidov et al., 1990). In agreement, perineuronal staining was absent in DE50-MD dogs, confirming that Dp427 is located around the soma and dendrites of canine neurons. Dystrophin immunolabelling was not detected in the granular neurons of the cerebellum, despite the detection of Dp140 mRNA by RNAScope ISH. This might result from low protein expression or high turnover (Dp427 is a highly stable protein, while the stability of Dp140 is less well characterised); techniques with greater sensitivity, such as electron microscopy with immunolabelling, might facilitate detection of low levels of dystrophin expression in certain regions of the canine bran. Our immunolabelling was further limited by the availability of antibodies that successfully labelled canine brain, and, hence, future work to develop a larger panel of canine-compatible antibodies would be beneficial.

Doorenweerd et al. (2017) used transcriptomic data from the Allen Human Brain and the BrainSpan atlases to identify expression of Dp427c and Dp427m mRNA in the adult human brain, most abundantly in the cerebral cortex. This aligns with our RT-qPCR data from the adult canine brain, whereby both Dp427c and Dp427m mRNA were detected in all studied regions, but most abundantly in the cerebral cortex. The Purkinje isoform, Dp427p, is expressed at very low levels in the adult human cerebellum but is not detected in the cerebral cortex (Doorenweerd et al., 2017). Again, this matched our findings in the canine brain, in which we identified low expression of Dp427p in the cerebellum only. The mouse had ∼10-fold higher expression of Dp427p in the cerebellum (Doorenweerd et al., 2017), which suggests that Dp427p might have a different function in mouse compared with human and dog. As implied by the name, this isoform is held to be expressed by Purkinje neurons; however, our ISH suggests that high levels of nascent transcripts are present within these neurons and that Purkinje neurons represent the principal source of full-length dystrophin expression within the cerebellum. Taken together, these data suggest that the majority of Dp427 expressed by Purkinje cells is not Dp427p; this isoform might instead either be expressed at a low level alongside other full-length isoforms or be restricted to a specialised subpopulation of Purkinje cells.

Dp140 mRNA and protein was detected in all studied regions of the adult canine brain, with highest expression in the cerebellum, as has been documented in the adult human brain (mRNA expression is 4-5× higher in the cerebellum compared with the cerebral cortex in both dog and human) (Doorenweerd et al., 2017) and adult murine brain (Lidov et al., 1995). Doorenweerd et al. (2017) identified high Dp140 expression in the early to mid-foetal stages in humans, with low expression from the late foetal stage onward, and we have shown that Dp140 is also the most abundant mRNA isoform in foetal canine brain at mid gestation (day 31 of 63) (Hildyard et al., 2020a), implying that similar regulatory mechanisms apply across species.

Dp71 was the most abundant isoform at the protein level, accounting for ∼80% of the total signal. This aligns with previous reports documenting abundant and relatively ubiquitous expression (Lidov et al., 1995; Waite et al., 2012; Doorenweerd et al., 2017; Tadayoni et al., 2012; Sarig et al., 1999). In contrast, Dp71 mRNA levels were more modest, comparable to those of Dp427c and Dp427m. There is no *a priori* reason why mRNA and protein should correlate closely, however, and as noted above, our RT-qPCR strategy does not distinguish between nascent and mature transcripts. The ∼16 h transcription time of Dp427 (Tennyson et al., 1995) (and ∼8 h time for Dp140) necessitates high levels of nascent transcripts, but production of Dp71 mRNA requires only ∼1 h, allowing supply to be more tightly coupled to demand. Nascent levels of this transcript might consequently be lower (supported by our ISH data; strong nuclear foci of 3′ probe are rarely observed). Transcription of Dp71 is, however, complicated by extensive alternative splicing that produces multiple isoforms, with 14 described to date. These isoforms show differing localisations within the cell and exhibit diverse functions (Naidoo and Anthony, 2020). The techniques used in this study do not distinguish Dp71 splice variants; future studies to elucidate the precise expression patterns of Dp71 in the canine brain might further refine our understanding of the extensive potential roles of this isoform.

The combination of approaches used here (immunoelectrophoresis, immunolabelling, RT-qPCR and ISH) reveals facets to dystrophin isoform expression that surpass those gained via any single technique alone. Despite this, the unique behaviour of this long gene remains challenging to study. Multiplex ISH allows isoform transcripts to be distinguished by length, but only by inference; binding of the 5′ probe is assumed to represent Dp427 (and the strong nuclear labelling associated with such expression is distinctive), and mid-probe and 3′ binding are a corollary of Dp427 expression, but this does not preclude the presence of additional shorter isoforms. It is not known whether a single cell can express multiple isoforms simultaneously, and, in cell-dense tissue that expresses multiple isoforms (such as the cerebellum), it is not always possible to assign specific isoforms to distinct cell populations. Furthermore, as noted above, the ISH approach cannot identify splice variants of Dp71, nor distinguish the three full-length Dp427 isoforms. In contrast, RT-qPCR can reveal which isoforms are present within a sample (and indicate the approximate levels of each), but cannot distinguish nascent from mature transcripts, nor where each isoform resides.

Dp427 protein was not detected in the DE50-MD dog brain. Our RT-qPCR data suggest a modest but consistent reduction (30-50%) in levels of Dp427 transcripts in DE50-MD brains (all three isoforms); these mRNAs carry a premature termination complex (PTC) (introduced by omission of exon 50) and so a plausible explanation is loss of mature transcripts to nonsense-mediated decay. Supporting this, the intense labelling of nascent Dp427 via ISH appears comparable between genotypes, suggesting that transcriptional initiation remains unchanged. Quantitative analysis of the RNAScope ISH data was not possible due to lack of access to age and breed-matched WT brain tissue (archived available brain tissue was stored in cryovials only and so not appropriate for sectioning). Future studies utilising appropriate matched samples from a range of ages would enable detailed quantitative analysis and comparison to RT-qPCR data. Previous work in DE50-MD skeletal muscle found no reduction in nascent transcription (via ISH or RT-qPCR), while mature transcripts (evaluated by RT-qPCR targeting 3′ sequence) were reduced to a similar degree as that shown here (Hildyard et al., 2020a). Interestingly, the same approach in *mdx* mouse skeletal muscle (Hildyard et al., 2020b) found stark reductions via both ISH and RT-qPCR, with mature sarcoplasmic transcripts essentially absent and even nascent transcription reduced. This may reflect inherent differences in dystrophin mRNA dynamics between species, and Dp427 might also be subject to more stringent nonsense-mediated decay in *mdx* mice. The site of mutation differs in these animal models (*mdx*, exon 23; DE50-MD, exon 50), and mRNAs carrying PTCs toward the 5′ terminus are typically more aggressively degraded than those lying 3′ (Carter et al., 1996; Supek et al., 2020).

Dp140 and Dp71 were detected in DE50-MD dog brains. The unique first exon of Dp140 resides in intron 44; Dp140 transcripts therefore normally carry exon 50 (and suffer loss of this exon in DE50-MD animals). Unlike other isoforms, however, the Dp140 first exon does not contain a translational start site; translation of Dp140 protein commences in exon 51, and thus exclusion of exon 50 results only in internal truncation of the long 5′ untranslated region (UTR). Consequently, Dp140 translation is not prevented by the mutation in exon 50. Taylor et al. (2010) compared DMD patients carrying a mutation in the promoter and/or coding region of the Dp140 isoform with those carrying a mutation in the extended 5′UTR and determined that FSIQ was lower in the former group. Our data provide the first demonstration at the protein level that mutations in the 5′ UTR do not prevent Dp140 expression. Interestingly, however, Dp140 protein expression was reduced in the DE50-MD brain compared with WT control brain, raising questions of whether this reduction might arise as a consequence of the genetic mutation disrupting transcriptional regulation of Dp140 or stability of the Dp140 transcript. We found no difference in Dp140 mRNA expression in DE50-MD dog brains compared with WT control brains, suggesting that transcription (initiation and survival of the transcript) might not be altered. However, our RT-qPCR targets the Dp140 first exon only; a significant fraction of the longer-isoform transcripts is present as nascent, rather than mature, mRNAs (Hildyard et al., 2020b) and thus not yet susceptible to most cellular mRNA turnover pathways. First exon expression might therefore not be truly representative of mature mRNA behaviour. Taylor et al. (2010) detected a lower FSIQ in patients with a mutation in the Dp140 5′ UTR compared with those with a mutation upstream of intron 44, and thus reduced Dp140 protein expression as a consequence of a mutation in the 5′ UTR may have consequences for brain function. Unlike Dp427, which localises to the synaptic membrane in neurons [with suspected roles in transmembrane transport, signal transmission and clustering of GABA_A_ receptors (Doorenweerd et al., 2017; Waite et al., 2012; Knuesel et al., 1999; Sekiguchi et al., 2009)], the roles played by Dp140 appear to be predominantly neurodevelopmental including axonal guidance, neuronal differentiation and transcription factor activity (Doorenweerd et al., 2017). Therefore, a reduction in Dp140 expression may influence neurodevelopment with consequences on subsequent cognitive function, and this could potentially play a role in the cognitive phenotype we have identified in the DE50-MD dogs.

A modest elevation in Dp71 protein expression was detected in the olfactory bulbs of DE50-MD dogs. This might represent a compensatory response to the loss of Dp427 and raises further questions over the potential for some degree of redundancy between dystrophin isoforms. Future work to identify precisely which splice variants of Dp71 are upregulated in the DE50-MD dog brain may reveal novel insights into their specific functions and potential for interaction, compensation and/or redundancy.

In conclusion, the canine brain shows dystrophin isoform expression comparable to that of the human brain but in contrast to that of the mouse brain (specifically Dp427p expression). The DE50-MD canine model of DMD is deficient in full-length dystrophin and displays a neurocognitive phenotype. As such, it is a translationally relevant pre-clinical model to study brain dystrophin function and to develop therapeutic strategies for the neurological sequelae of DMD. Furthermore, the precise mechanisms by which dystrophin deficiency compromises brain function remain to be elucidated, and the DE50-MD dog might provide a key tool in this endeavour; characterisation of the function and downstream signalling effects of each isoform in the canine brain will be an important focus of future studies.

## MATERIALS AND METHODS

### Animal husbandry

Dogs (*Canis familiaris*) were group housed (12 h light/dark cycle; 15-24°C) at the Royal Veterinary College, in a dedicated canine facility with large pens (minimum of 2×4.5 m), access to outdoor runs, grass paddocks and different types of enrichment items, conditions that exceed the minimum stipulated by the UK Animal (Scientific Procedures) Act 1987. Carrier female Beagle (RCC strain)-cross dogs (F3 generation) derived from an original founder female carrier (Bichon-Frise cross Cavalier King Charles Spaniel) were mated with male Beagles (RCC strain) to produce WT, carrier and DE50-MD offspring. Adult dogs were group housed until females were close to whelping; thereafter, pregnant females were housed individually and allowed to whelp naturally. All puppies within a litter (including those on trials) were kept with their mother in a large pen to enable nursing, with access to a bed under a heat lamp (∼28°C). From 4 weeks of age, puppies were introduced to *ad libitum* puppy feed (Burns) until weaning at 12 weeks. Thereafter dogs received two feeds daily and *ad libitum* water. All animals followed a comprehensive socialisation programme with daily human interactions and were acclimatised to routine procedures. Welfare assessments were conducted twice daily. All work was conducted under UK Home Office Project Licence (numbers PPL 70-7515 and P9A1D1D6E) and approved by the local Animal Welfare Ethical Review Board.

### Cognitive testing

A range of behavioural tests was piloted in the DE50-MD dogs and WT controls. Muscle pathology in the DE50-MD dog is typical for DMD and is characterized by variable myofibre size, areas of degeneration (necrosis and phagocytosis) regeneration, fat deposition and atrophy (Hornby et al., 2021; Walmsley et al., 2010). To minimise influence of the dogs' physical impairment in tests of cognitive function, we selected tests of short duration that were independent of strength or physical exertion (González-Martínez et al., 2013; Siwak et al., 2001; Protopopova et al., 2014). Tests selected for the final battery are reported below. All testing was conducted in age-matched groups by one investigator and an assistant. Sample size calculations were not performed as the sample was limited to dogs available in the colony through a separate natural phenotype study; no dogs were bred specifically for this study and all work was conducted in alignment with other studies in accordance with the institute's policy to minimise experimental dog numbers. In total, eight male DE50-MD dogs and six male age-matched WT controls were available for testing. A further two male WT stud dogs were included in the response to novelty (mirror) testing (age 25 and 29 months) as this was performed in kennel groups. Age at which each test was performed and number of dogs tested is detailed in Table S1. Individual tests were conducted in all dogs on the same day, with at least a 7-day period between subsequent tests. Two DE50-MD dogs were unavailable for the response to cognitive enrichment and acquisition/extinction learning due to concerns over respiratory status, recent general anaesthesia or use in a concurrent experiment. All testing was video recorded for subsequent analysis by an investigator blinded to genotype and hypothesis to test.

#### Response to novelty

Dogs were assessed for response to (1) a novel olfactory cue and (2) a mirror. In humans, enhanced response to novelty has been linked to better performance on neuropsychological tests, especially those involving attention/executive functions (Daffner et al., 2006), while ageing and declining cognitive function have been associated with reduced response to novelty (Fabiani et al., 1998; Walhovd and Fjell, 2001), in which the prefrontal cortex appears to play a central role (Daffner et al., 2000).

For testing response to a novel olfactory cue, dogs were tested individually and allowed into the study room, in which a novel olfactory cue (ball impregnated with pig scent) was positioned in the centre of the floor. The dog was video recorded for the subsequent 3 min. Time to detect the olfactory cue (determined by making visual contact and/or approaching the cue), the duration of time for which the dog investigated the cue (sniffed, pawed or other physical contact), the number of times the dog returned to the cue and the number of times the dog marked (urinated on) the cue were recorded.

For testing response to a mirror, an acrylic mirror (30×42 cm) was placed at the front of the home kennel. WT dogs were kennelled in two groups (consisting of five and three dogs). The DE50-MD dogs were kennelled in three groups (three, three and two dogs). Dogs were video recorded in their kennel for 10 min after positioning the mirror. Data collection began at the time the mirror was placed in front of the kennel. Interaction with the mirror was defined as looking towards the mirror from any position in the kennel and could include vocalising, pawing, sniffing or jumping at the mirror. Recorded variables included total time spent interacting with the mirror, number of returns to the mirror, jumping up on the kennel door and duration of vocalisation (barking and growling at the mirror).

#### Response to a cognitive enrichment

Dogs were individually allowed into the study room, in which a cognitive enrichment (a puzzle toy: Nina Ottosson by Outward Hound Dog Smart Orange Interactive Treat Puzzle Dog Toy) was presented in the centre of the floor to assess problem-solving ability, motivation and attention. The puzzle consisted of nine small removable white plastic pieces in an orange frame, with a single food treat positioned below each of the nine removable white plastic pieces. Access to a treat required the dog to displace the overlying white plastic piece by gently pushing with the nose or paw, and so required minimal to no exertion or physical strength. Dogs were video recorded with the puzzle for 5 min. If the dog failed to obtain a treat after 2.5 min of recording, one plastic piece was removed by the investigator to reveal the underlying treat. If the dogs successfully obtained all nine treats within 5 min, the recording was stopped. Recorded variables were time to detect the puzzle toy (defined as visualisation and approach to investigate), as well as duration of time interacting with the puzzle (defined as sniffing, exploring, pawing or otherwise contacting the puzzle), time to obtain first treat, time to obtain all treats (where applicable) and total number of treats obtained.

#### Acquisition and extinction learning

Acquisition and extinction of a previously rewarded response was used to assess cognitive performance, as previously reported in dogs (Lazarowski et al., 2014). During the acquisition phase, the investigator stood facing the dog with their left hand at hip height and palm facing the dog. A food reward (dog treat) was offered to the dog in the palm of the left hand and repeated a further four times to ensure that the dog had associated the left hand with the reward. Thereafter, the investigator offered their empty left hand to the dog; every time the dog touched the investigator's hand with its nose, a reward was offered, up to a total of 20 treats. If 15 s passed with no response from the dog, the investigator attempted to re-engage the dog's attention by re-presenting their left hand while calling the dog's name. If still no response was generated, a treat was placed in the left hand. If the dog still did not respond, a 30 s rest period was allowed and the treat reoffered in the left hand. Failure to generate the nose touch response after the 30 s rest period resulted in termination of the experiment and the dog being recorded as a non-responder. If attention was regained but the dog subsequently again lost attention, the above step was repeated. Following a third loss of attention, the experiment was concluded, and the dog was recorded as a non-responder. Recorded variables included the time taken to the first successful nose touch (s), the duration (s) and percentage of total recording time the dog remained focused on the task, the number of behavioural changes (e.g. focused on task to sniffing or exploring) observed during the recording and the number of non-responders.

Extinction was assessed once a total of 20 treats had been received (consecutively or following attempts to regain attention) in the acquisition phase. No further treats were offered to the dog. When the dog contacted the investigator's hand with his nose, the investigator withdrew the left hand behind the back briefly before replacing it at hip height, and no treat was delivered. The extinction phase ended when the dog did not attempt to contact the investigator's hand for 1 min. Recording variables included the duration and percentage of time the dog remained focused on the extinction phase, the number of nose touches in the extinction phase and the number of behavioural changes observed.

#### EBR

EBR has been used as an indicator of dopaminergic function (Lawrence et al., 1991; Mavridis et al., 1991), with reduced EBR reported in a range of neurological disorders including ADHD (Konrad et al., 2003) and epilepsy (Caplan et al., 1998). Dogs were gently restrained by an assistant and the eyes were video recorded for 60 s. The total number of blinks (defined by closing of the eyelids to cover at least 50% of the visible corneal surface) in 60 s was recorded using slow motion replay (0.5× normal speed).

### Tissue collection

Brain tissue was collected from five DE50-MD dogs and five WT littermate controls during a natural history study. Dogs were euthanised by pentobarbital intravenous injection, and the brain was harvested and dissected within 60 min. Approximately 1 cm^3^ samples of regions of interest of the canine brain were collected; samples for protein/RNA isolation were placed in cryovials, snap frozen in liquid nitrogen and subsequently pulverised under liquid nitrogen in a pre-chilled mortar and pestle to produce frozen tissue homogenates. In addition, WT mouse (*Mus musculus*, cranialis tibialis) and WT dog (vastus lateralis) skeletal muscle was processed identically to provide positive control tissue for protein analysis. For DE50-MD dogs only, brain samples for cryosectioning were mounted on cork discs and frozen under liquid-nitrogen-cooled isopentane to preserve tissue morphology. The Royal Veterinary College's Companion Animal Brain Bank provided WT male dog brain samples for cryosectioning, as archived samples from the natural phenotype study were not available (*n*=2, 2-year-old French bulldog and 1-year-old Cockapoo). All frozen samples were stored at −80°C until use. Remaining brain tissue (DE50-MD and Companion Animal Brain Bank WT) was fixed in 4% paraformaldehyde prior to processing to paraffin wax.

### Capillary immunoelectrophoresis

Approximately 100 mg of frozen tissue powder (see above) was mixed at a 1:4 ratio (w/v) with SDS lysis buffer (10% SDS, 62.5 mM Tris-HCl, 1 mM EDTA pH 6.8) containing protease inhibitors (Complete Mini, Roche). Lysates were first homogenised via plastic micropestle and then passed through syringe needles (21 gauge, then 25 gauge). Any remaining insoluble material was removed by centrifugation (8000 ***g***, 5 min). Protein content of lysate was determined via DC protein assay (Bio-Rad), and lysates were either used immediately or frozen in 10 μl aliquots and stored at −80°C.

Dystrophin protein expression was evaluated via capillary immunoelectrophoresis (ProteinSimple Jess), using a C-terminal antibody (Abcam154168, 1:250) and anti-mouse HRP (ProteinSimple). Lysates were loaded at 0.1 μg/μl, and all dystrophin signal intensities were normalised to total protein load (determined via Total Protein RePlex assay as per the manufacturer's instructions). Relative abundance was calculated for the full data set by using the maximum value across all isoforms.

### Immunofluorescence

Frozen brain samples were cryosectioned at −25°C to 12 μm thickness using an OTF5000 cryostat (Bright) and mounted on glass slides (SuperFrost, VWR). Serial sections were collected, and slides were dried at room temperature for 1 h before storage at −80°C until use.

For staining, slides were removed from −80°C and allowed to equilibrate to room temperature (∼15 min), then fixed in a 1:1 mix of acetone and methanol for 15 min at −20°C. Slides were washed in phosphate buffered saline (PBS), before blocking for 1 h at room temperature in 5% bovine serum albumin with 0.3 M glycine and 2% triton. Primary antibodies were diluted in blocking solution and applied overnight at 4°C (see Table S2 for dilutions). Slides were washed three times in PBS prior to application of secondary antibodies (Invitrogen goat anti-mouse 488, goat anti-rabbit 594) at 1:750 dilution. Slides were incubated at room temperature for 2 h, followed by incubation in Hoechst solution (1:2000 dilution) for 10 min. Following a final three washes in PBS, slides were mounted with Hydromount (National Diagnostics).

### RT-qPCR

#### RNA isolation

Approximately 100 mg of pulverised frozen tissue was placed directly into 1 ml TRIzol reagent (Invitrogen). RNA was extracted following the manufacturer's instructions, with inclusion of a second 1:1 chloroform extraction following phase separation and addition of glycogen at 10 mg/ml during precipitation to maximise RNA yield, as previously described (Hildyard et al., 2019, 2018). RNA purity was assessed by spectrometry (Nanodrop ND1000), with samples exhibiting significant guanidium carryover (260/230, <1.7) cleaned with a second isopropanol precipitation step.

#### cDNA synthesis

cDNA was prepared using the RTnanoscript2 kit (PrimerDesign) using random nonamer and oligodT priming, with 1.6 μg of total RNA per 20 μl reaction. Following synthesis, cDNA samples were diluted (1/20) to minimise PCR-inhibitory contributions from cDNA synthesis buffer components, giving a final cDNA concentration of ∼4 ng/μl (assuming 1:1 conversion of RNA to cDNA).

#### Quantitative PCR (qPCR) and analysis

qPCR reactions were performed in triplicate with 2 μl cDNA per well (∼8 ng), using PrecisionPLUS SYBR Green Mastermix (PrimerDesign). Primers to *SDHA*, *UBC* and *YWHAZ* [previously validated reference genes for canine brain (Crawford et al., 2021)] were taken from the *C. familiaris* geNorm kits (PrimerDesign) and are proprietary. qPCR primers to canine dystrophin were used as previously published (Hildyard et al., 2020a). PCR was conducted in a CFX384 light cycler (Bio-Rad) in a three-step PCR (95°C, 15 s; 60°C, 20 s; 72°C, 20 s for 40 cycles) with subsequent melt curves performed for all reactions. All primer pairs gave sharp, single-amplicon products and single melt peaks. Quantification cycle (Cq) values were determined by regression. Relative quantities (RQ) were calculated for the full data set by using the minimal Cq value across all isoforms to enable assessment of individual isoform expression relative to total dystrophin expression (sum of expression of all isoforms). RQ data were then normalised to the geometric mean of three previously validated reference genes: *SDHA*, *UBC* and *YWHAZ* (Crawford et al., 2021).

### RNAScope ISH

#### Probe design

Twenty ZZ RNAScope probes (ACDBio) designed to mouse dystrophin sequence (accession number NM_007868.6) were used to examine canine dystrophin mRNAs as reported previously (Hildyard et al., 2020a,b). Use of 20 ZZ oligonucleotide probe pairs in series enables targeting of ∼1000 bases of sequence. The catalogue probe (Mm-Dmd, Cat. No. 452801) in the C1 channel detects residues 320-1295 (exons 2-10). The custom probes in the C2 (Mm-Dmd-O1-C2, Cat. No. 529881-C2) and C3 channels (Mm-Dmd-O2-C3, Cat. No. 561551-C3) recognise residues 9581-10846 (exons 64-75) and residues 6692-7764 (exons 45-51), respectively. C1 (5′ probe) labels full-length dystrophin isoforms only (Dp427c, m and p), the C2 (3′ probe) labels all dystrophin isoforms, and C3 (mid probe) labels Dp427, Dp260 (not expressed in brain) and Dp140 (Fig. 1). Presence of 3′ probe alone indicates expression of Dp71 (Dp116 and Dp40 have not been identified in any mammalian brain tissue). Dystrophin sequence is highly conserved, and the regions covered by these 20 ZZ probes show a high identity between mouse and dog (89.27%, 86.39% and 93.52% identical for 5′, middle and 3′ probes, respectively), sufficient for probe binding as reported previously (Hildyard et al., 2020a). Positive control probes to POLR2A (NM_009089.2, residues 2802-3678), PPIB (NM_011149.2, residues 98-856) and UBC (NM_019639.4, residues 36-860) were used to confirm preservation of sample RNA, while negative control probes to bacterial DapB (EF191515, residues 414-862) were used to examine possible non-specific labelling.

#### Sample preparation

Formalin-fixed, paraffin-embedded (FFPE) brain samples were cooled on ice and sectioned at 4 μm thickness using a microtome (Leica Biocut), then floated in a water bath at 48°C and mounted on Superfrost slides. Slides were dried at 37°C overnight and stored at room temperature in sealed containers until use. Sections were treated according to the RNAScope multiplex fluorescent reagent kit v2 (ACDBio) protocols for FFPE, with target retrieval using the manufacturer's ‘alternative method’: slides were immersed slowly in target retrieval buffer (held at a gentle boil) for 15 min, before cooling directly into room temperature distilled water, followed by ethanol dehydration.

#### RNAScope multiplex assay

Multiplex assays were performed as recommended by the RNAScope multiplex fluorescent reagent kit v2 protocols (ACDbio). The following probe mixes were used: (1) RNAScope 3-plex positive control probe set (320881): POLR2A, PPIB and UBC (C1, C2 and C3 channels, respectively); (2) RNAScope 3-plex negative control probe set (320871): bacterial DapB (in C1, C2 and C3); (3) RNAScope mouse dystrophin probe set: Mm-Dmd (452801), Mm-Dmd-O1-C2 (529881-C2) and Mm-Dmd- O2-C3 (561551-C3) (C1, C2 and C3 probes to 5′, 3′ and mid sequence of Dp427 transcript, respectively).

After completion of the RNAScope labelling, slides were incubated in Hoechst (1:2000 dilution in wash buffer, 5 min), mounted in Prolong Gold (Thermo Fisher Scientific) and air dried overnight (protected from light). Fluorophores were assigned as follows: 5′ probe (C1), TSA-Cy3; middle probe (C3), TSA-Opal520; 3′ probe (C2), TSA-Cy5.

### Imaging

A DM4000B upright microscope was used for image capture with samples illuminated using an EBQ100 light source and A4, N3, L5 and Y5 filter cubes (Leica Microsystems) and an AxioCam MRm monochrome camera with Axiovision software (Version 4.8.2, Carl Zeiss). Images shown in figures have been adjusted for clarity using the window/level tool of ImageJ. Quantification of immunolabelling was performed on five images (technical replicates), taken with the 40× objective, per brain region (cerebral cortex, cerebellum and brainstem) per animal (biological replicate). Quantification was performed using the Cell Count function in the Fiji distribution of ImageJ by a single investigator blinded to genotype and hypothesis to test.

### Statistical analysis

Statistical analysis was performed using GraphPad PRISM 9.0. Data were assessed for normal distribution by evaluation of histograms and the Shapiro–Wilk normality test. Data are presented as mean±s.d. where a normal distribution was identified, otherwise data are shown as median and range (all cognitive testing data presented as median and range for consistency). Cognitive testing and immunofluorescence data were assessed using the Mann–Whitney test for continuous data or Fisher's exact test for categorical data. RT-qPCR and capillary immunoelectrophoresis data showed a normal distribution, and analysis was performed with repeated measures one-way ANOVA with Sidak's multiple comparisons test with significance set at *P*<0.05.

## Supplementary Material

Supplementary information
