# Supplementary material for: Validation of DE50-MD dogs as a model for the brain phenotype of Duchenne muscular dystrophy
Source: Dis Model Mech. 2022 Mar 2;15(3):dmm049291. doi: 10.1242/dmm.049291 (PMC8906169; doi:10.1242/dmm.049291)
Supplement: Supplementary information [file dmm-15-049291-s1.pdf]

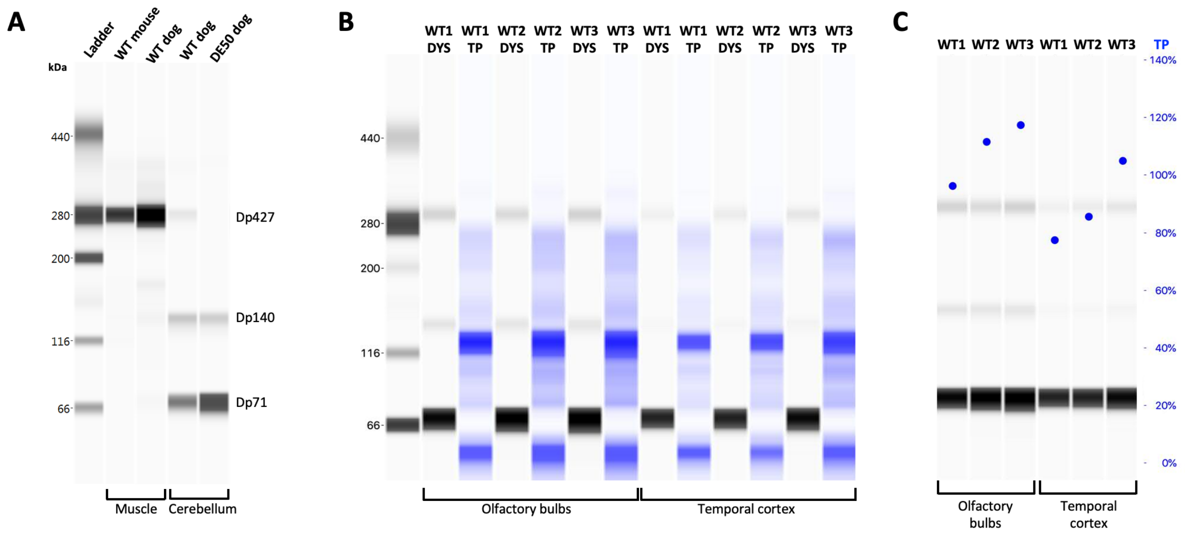

Fig. S1. Capillary immunoelectrophoresis for dystrophin with total protein assay. WT mouse (cranialis tibialis) and dog (vastus lateralis) skeletal muscle express only full-length dystrophin, confirming that the band detected at ~280kDa is Dp427, as has been previously reported and likely a consequence of the molecular weight ladder used, which underestimates molecular weights above 280 kDa (Beekman et al., 2018). B) Total protein loading (TP) per lane is shown adjacent to dystrophin labelling of each sample. C) Samples with total protein overlay shown as a blue circle.

Disease Models & Mechanisms • Supplementary information

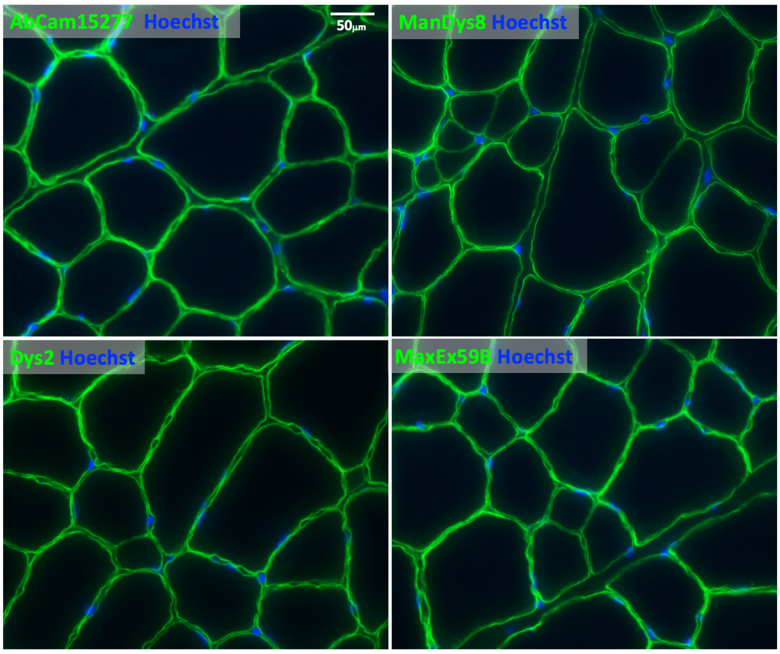

Fig. S2. WT canine skeletal muscle immunolabelling with various dystrophin antibodies (positive control). Dense dystrophin-positive staining of the myofibre membrane is seen with all tested antibodies.

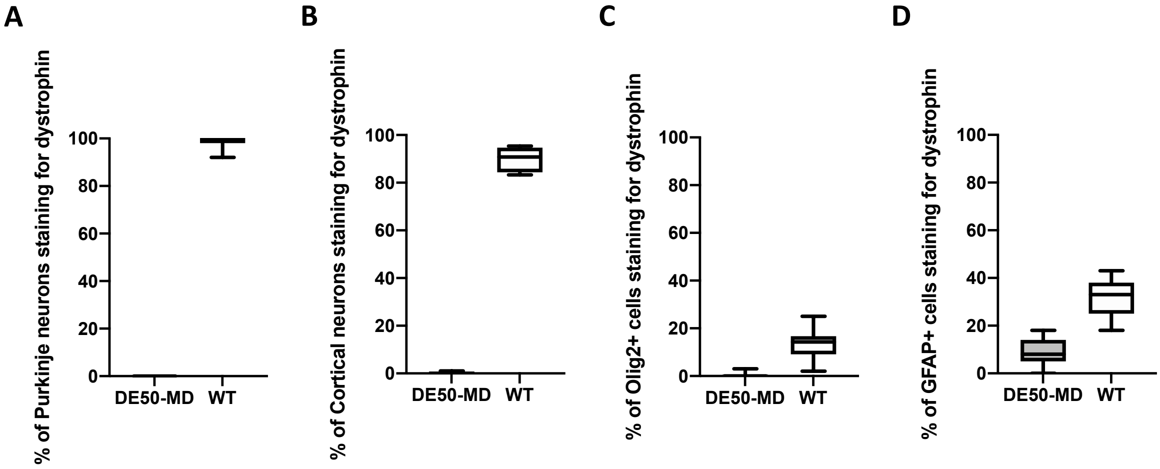

Fig. S3. Percentage of neurons, oligodendrocytes and astrocytes immunolabelled for dystrophin in the canine brain. A) Dystrophin positive staining was detected in the majority of Purkinje neurons in WT canine cerebellum but was absent in DE50-MD dogs. B) Dystrophin positive staining was detected in the majority of cerebral cortical neurons in WT canine brain. C) Dystrophin positive staining was detected in Olig2+ cells in WT canine brain. D) Dystrophin positive staining was detected in GFAP+ astrocytes in WT and, to a lesser extent, DE50-MD dog brains (n=2 WT and 3 DE50-MD dogs, 100-200 cells counted per animal).

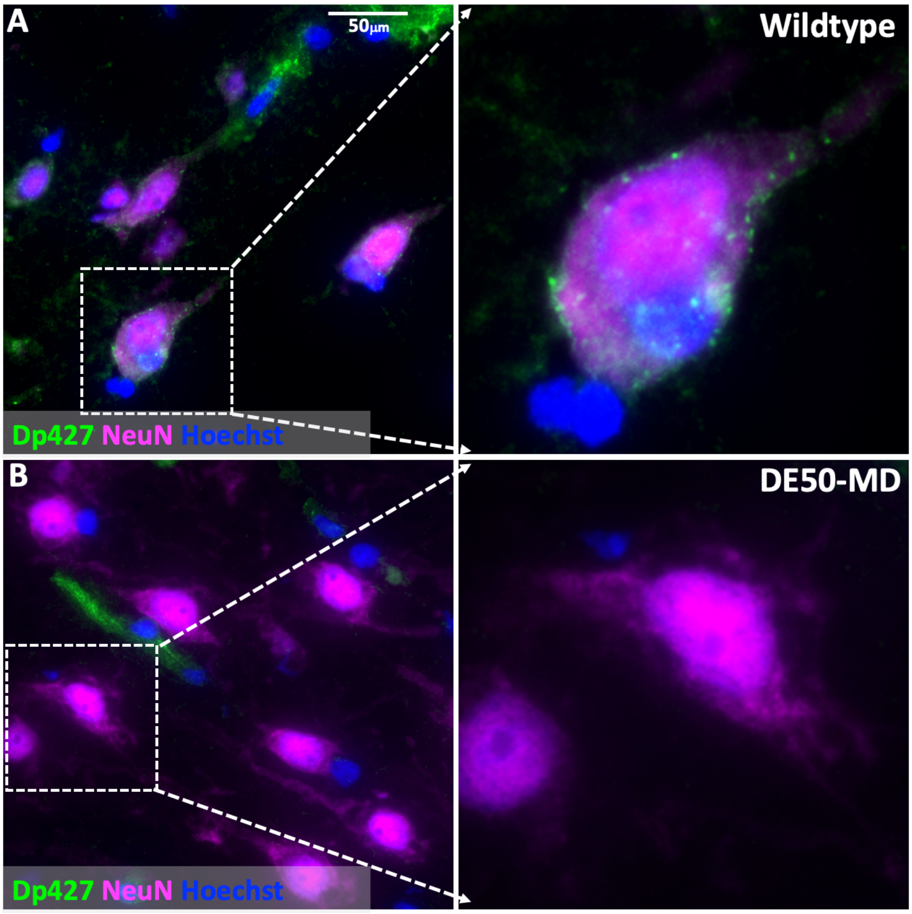

Fig. S4. Immunolabelling of dystrophin in the canine cerebral cortex with co-labelling for NeuN as a neuronal marker. A) Occipital cortex of a WT dog: punctate dystrophin-positive staining is detected around the cell body of cortical neurons. B) Occipital cortex of a DE50-MD dog: no dystrophin-positive neuronal staining is detected. (Dystrophin detected with ManDys8 antibody, which detects Dp427 and Dp260 (not expressed in brain).

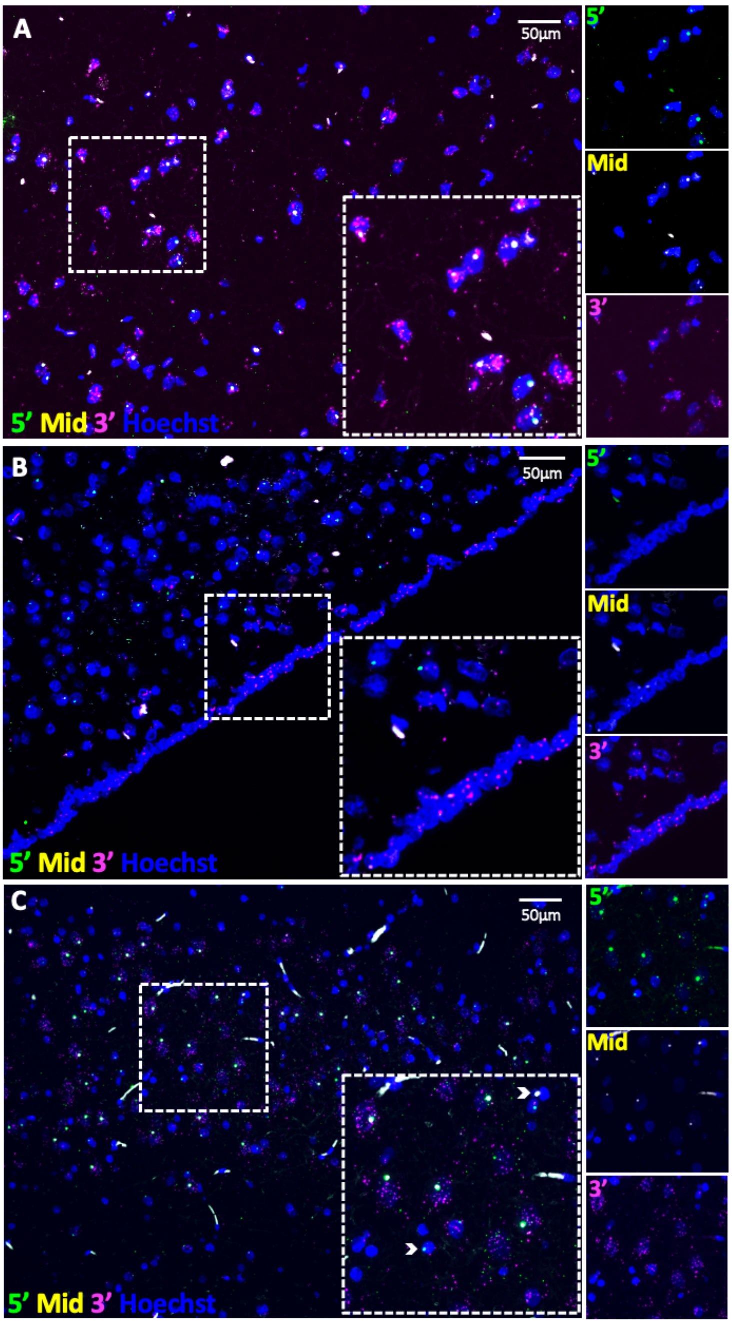

Fig. S5. RNAScope in situ hybridization reveals Dp427, Dp140 and Dp71 mRNA expression in the DE50-MD canine occipital cortex, ependyma and CA2/3 regions of the hippocampus. A) 5' probe binding is detected in neurons of the cerebral cortex, consistent with Dp427 expression. B) Ependymal cells lining the lateral ventricle show abundant 3' probe binding, consistent with Dp71 expression. Occasional ependymal cells show a small focus of mid transcript probing, suggesting low levels of Dp140 expression. C) Neurons in CA2/3 of the hippocampus show prominent 5', mid transcript and 3' probe binding, with large foci of 5' binding at the dystrophin locus, consistent with Dp427 expression. Some small dense nuclei (arrowheads), likely representing glia, also show binding of all 3 probes.

Disease Models & Mechanisms • Supplementary information

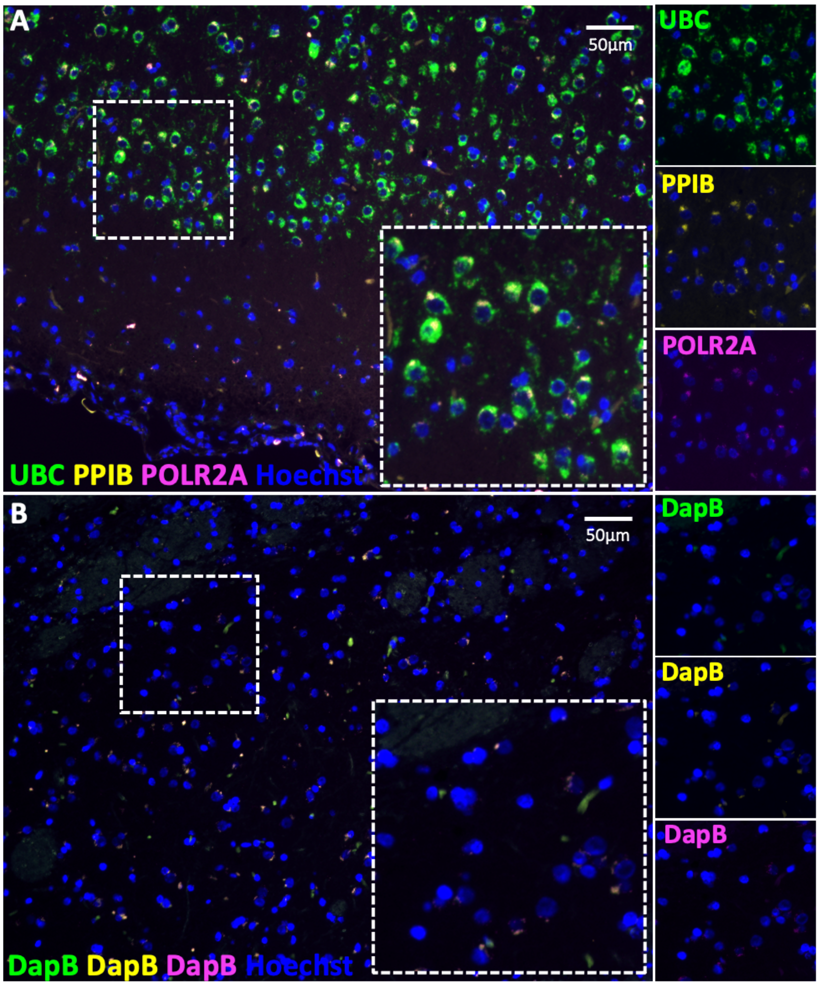

Fig. S6. RNAScope positive and negative controls. Positive control probes to (mouse POLR2A, PPIB and UBC label canine cerebral cortex (A). Negative control probes (bacterial DapB) do not label canine cerebral cortex (B).

Disease Models & Mechanisms • Supplementary information

Table S1. Age of WT and DE50-MD dogs at the time of each behavioural test. Age is shown as median (range).

Behavioural test	No. of WT dogs tested	Age (months) of WT dogs at time of testing	No. of DE50-MD dogs tested	Age (months) of DE50-MD dogs at time of testing
Response to novelty (olfactory cue)	6	8 (6-18)	8	12 (8-18)
Response to novelty (mirror)	6 (+ 2 stud males)	8 (6-18)	8	12 (8-18)
Response to a cognitive enrichment	6	9 (7-19)	6	13 (9-19)
Acquisition and extinction learning	6	11 (9-21)	6	15 (11-21)
Eye blink rate	6	13 (11-23)	8	17 (13-23)

Table S2. Details of the primary antibodies used. (GFAP: Glial fibrillary acidic protein, NA: not applicable, DSHB: Developmental studies hybridoma bank). Dystrophin antibodies found to give reliable staining in canine brain are shown in bold.

Antigen	Dystrophin isoforms detected	Species raised	Type	Manufacturer & Catalogue number	Dilution
Dystrophin (C terminus)	All	Mouse	Monoclonal	Novocastra (Dys1CE) (Clone DY4/6DE)	1:10
Dystrophin (C terminus)	All	Mouse	Monoclonal	Novacastra (Dys2CE) (Clone DY8/6C5)	1:10
**Dystrophin (C terminus)**	**All**	**Rabbit**	**Polyclonal**	**AbCam (Ab15277)**	**1:200**
Dystrophin (Exon 77)	All	Mouse	Monoclonal	DSHB (Mandra1(7A10))	1:20
**Dystrophin (Exons 31-32)**	**Dp427, Dp260**	**Mouse**	**Monoclonal**	**DSHB (ManDys8(8H11))**	**1:20**
**Dystrophin (Exon 59)**	**Dp427, Dp260, Dp140**	**Mouse**	**Monoclonal**	**DSHB (ManEx59B (4E10))**	**1:20**
Dystrophin (Exons 10-11)	Dp427	Mouse	Monoclonal	DSHB (ManEx1011C (4F9))	1:20
NeuN	NA	Rabbit	Monoclonal	AbCam (Ab177487) (EPR12763)	1:1000
GFAP	NA	Mouse	Monoclonal	Millipore (MAB360) (Clone GA5)	1:3000
Olig2	NA	Rabbit	Polyclonal	Millipore (AB9610)	1:500
